# Strengthening the mpox response: how do we balance pragmatism and equity in resource-constrained settings?

**DOI:** 10.7189/jogh.15.03036

**Published:** 2025-11-14

**Authors:** Virgil K Lokossou, Kehinde O Ogunyemi, Aishat B Usman, Simplice Kamdem, Césaire D Ahanhanzo, Felix Agbla, Melchior A Aïssi, Issiaka Sombié

**Affiliations:** 1 West African Health Organization, Bobo, Dioulasso, Burkina Faso; 2 Hubert Department of Global Health, Emory University, Atlanta, USA; 3 Department of Epidemiology and Biostatistics, University of Georgia, Athens, USA; 4 Regional Head Office, USAID/West Africa, Accra, Ghana; 5 WorldBank/Western and Central Africa, Health, Nutrition, and Population Global Practice HAWH3, Cotonou, Benin

## Abstract

Mpox, previously neglected as an endemic zoonotic disease in West and Central Africa, has become a significant global threat following its second declaration as a public health emergency of international concern. With the growing number of cases, the region faces several challenges, particularly inadequate surveillance and diagnostics, and a lack of vaccine access. Drawing on lessons from public health emergency response in the Economic Community of West African States region, we aim to inspire a new vision with health emergency preparedness and response framework transformed for implementing mpox response to improve people’s health and strengthen national, regional, and global health security. We provide insights into how pragmatism and equity considerations can guide short- and long-term overarching strategies and actions to achieve this vision, particularly in low-resource settings. We also identify sustained political commitments and investments at all levels of health governance as cross-cutting imperatives for controlling the outbreak. Specifically, vaccine access strategies (phased rollout, community-based distribution models and dose-sparing administration) and surveillance and diagnostics strategies (stigma-sensitive risk communication and community engagement, increased health workers and volunteers’ training, and decentralised monitoring and testing) were highlighted as critical for optimal control. Based on this, we call for a strengthened mpox response that leverages local networks (*e.g.* community-based organisations, non-governmental organisations, and groups caring for men who have sex with men (MSM)/sex workers networks), the private sector, and digital solutions. To improve the coverage, impact, and sustainability of health and social interventions for this response, we emphasise a focus on at-risk populations (young children, MSM/sex workers, health workers/volunteers, contacts of cases), vulnerable populations (people living with HIV, people on immunosuppressive therapy, and pregnant women), and underserved communities (rural areas, hard-to-reach locations, and humanitarian settings), alongside a reformed global health architecture and strengthened regional and national health systems.

Mpox, formerly monkeypox, is an infectious zoonotic disease caused by the monkeypox virus (MPXV) characterised by skin rash, often accompanied by fever, muscle aches, headache, and other symptoms lasting 2–4 weeks [1]. It was responsible for a large mpox outbreak between July 2022 and July 2023 that affected more than 110 countries. This resulted in 85 473 confirmed cases and 89 deaths (case fatality rate of 0.1%) globally, and 401 cases in Africa, specifically, of which 202 occurred in Central Africa and 194 in West Africa [2,3].

The Africa Centres for Disease Control and Prevention (Africa CDC) recently reported increases of 160% and 19% in the numbers of mpox cases and deaths in the region, respectively, in 2024 compared to 2023, with a similar trend also observed between 2023 and 2022 [4]. This upsurge of cases and the potential public health risk, alongside other factors, prompted the Africa CDC and the World Health Organization to declare a public health emergency of international concern on 13 and 14 August 2024, respectively [4,5].

As of 6 September 2024, 24 851 cases (19 302 suspected, 5549 confirmed, case fatality rate of 2.6%) and 643 deaths were reported in 14 African countries, representing a 64% increase in confirmed cases over two weeks from the last epidemiological update with the highest number of cases reported in the Democratic Republic of the Congo [6,7]. While this figure shows that the number of cases is rapidly growing – and with even higher stakes, given evidence of the sustained transmission of a recently identified clade Ib strain which is thought to be more transmissible and severe than previous strains [8] – it might not represent the true burden of the disease due to potential biases from differences in underascertainment and underreporting. The common knowledge that underascertainment biases are often driven by social factors (*e.g.* undetected symptomatic cases from limited awareness on mpox symptoms or inadequate case definition, undetected symptomatic and asymptomatic cases from lack of access to diagnostic interventions from financial or geographical constraint) and underreporting by technical factors (*e.g.* undiagnosed cases from limited availability or accuracy surveillance and diagnostics, unregistered cases from lack of error-free data entry/exchange infrastructure or low data quality check), all suggest that we are dealing with a complex problem.

The mpox response has not only being challenging because of its endemicity in 12 African countries – West Africa (Benin, Ghana, Cote d’Ivoire, Liberia, Nigeria, and Sierra Leone) and Central Africa (Cameroon, Central African Republic, the Democratic Republic of the Congo, Gabon, the Republic of the Congo, and South Sudan – but also due to several other interconnected factors. These include the mpox disease rapidly changing epidemiological, virological, and clinical patterns; decades-long periods of declining population immunity from smallpox vaccination cross-protection and changes in populations structure following its eradication in 1980; low political commitments and lack of dedicated national funding mechanism for health emergencies; suboptimal surveillance and diagnostics and vaccine access; chronic underinvestment in the disease research and development; and existing chronic problems of political instability and insecurity, points of entry porousness, shortage of health (health care and public health) workers, multiple and concurrent disease outbreaks, and stakeholders power dynamics and competing interests, all presenting a significant cross-border and global spread risk [9–18].

To this end, there is considerable reason to believe that there are still many uncertainties and unknowns about mpox, such as its natural reservoirs, evolutionary pattern, and the role of ecological changes (*e.g.* agricultural expansion, urbanisation, climate change) in its transmission and severity [1,11,19]. The global health community cannot afford to repeat the mistakes of past responses to mpox and the COVID-19 pandemic that were characterised by cycles of reactive response, neglect, and complacency.

In this viewpoint, we discuss the burden, challenges, and strategies and actions for mpox response in West Africa and beyond through a pragmatic and equity lens. We detail the significance of this issue and the framework underlying this analysis on p. 2 and 3, and in Figure S1 in the **Online Supplementary Document**, respectively [20,21].

## RAPID SITUATIONAL ASSESSMENT OF MPOX RESPONSE: INSIGHTS FROM THE ECONOMIC COMMUNITY OF WEST AFRICAN STATES (ECOWAS) REGION

### Burden

**Figure 1:**
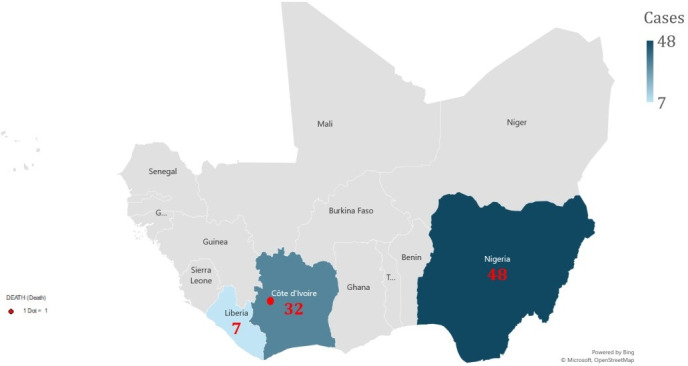
Geographic distribution of confirmed mpox cases in West Africa, as of 2 September 2024 [24].

According to our analysis on behalf of the West African Health Organization (WAHO), as of 2 September 2024, the risk for mpox in West Africa was considered moderate, consistent with the WHO risk assessment report (Figure S2 in the **Online Supplementary Document**) [22–24]. A total of 87 confirmed cases and a single death (case fatality rate of 1.2%) have been reported in the region, affecting Cote d’Ivoire, Liberia, and Nigeria, and the clade II viral strain has been implicated in the transmission [24]. This figure represents a 19% increase in confirmed cases over three weeks from the last epidemiological update, with the highest number of cases reported for Nigeria (**Figure 1**) [24].

The distribution of cases and deaths by high-risk groups – at-risk populations (*i.e.* young children, MSM/sex workers, health workers/volunteers, contacts of cases), vulnerable populations (people living with HIV, people on immunosuppressive therapy, pregnant women), and underserved communities (rural areas, hard-to-reach communities, humanitarian settings) – were not fully described at the time of writing this viewpoint due to incomplete or delayed case reporting from the ECOWAS Member States. However, based on available literature [6,25], the target population that is likely to be most at risk are young children (under 15 years), suggesting the need for whole-of-society and risk-based response interventions to address two key challenges, which we outline below.

### Inadequate surveillance and diagnostics

Surveillance and diagnostics remain the foundation of national, regional, and global health security [2,4,11]. They are critical for assessing the true burden of mpox through early outbreak detection and response, reliable case detection, and timely case reporting. While existing integrated disease surveillance and response systems that incorporate both indicator-based and event-based surveillance approaches are not without some strengths, such as improved outbreak detection and notification timeliness, standardised case definition adoption, and health workforce training [2], the mpox response could be challenged by several weaknesses, particularly in underserved communities. Examples of these weaknesses include [4,11,12,26,27]:

− inadequate training of health workers, community volunteers, and laboratory personnel on mpox surveillance and diagnostic competencies, such as risk communication and community engagement, infection prevention and control, screening, sample collection, transport and testing, and biosafety;

− lack or fragmented use of event-based surveillance system in mass gatherings for supporting early outbreak detection; lack of consistency in the use of standardised case definitions;

− uneven distribution of health facilities, resulting in prolonged travel time for suspected cases to access health facilities for screening, as well as for their skin lesion samples to be transported to laboratories for testing;

− limited availability of laboratories with real-time polymerase chain reaction (PCR) tests and reagents for confirmatory diagnosis and/or genomic sequencing of MPXV;

− instability of electric power supply for optimal refrigeration or freezing of clinical samples;

− suboptimal digitisation of surveillance and diagnostics databases for streamlined and real-time data collection, case reporting, and disease burden and response outcomes assessment.

These submissions are supported by emerging evidence on mpox response in the region, where 10 (71.4%) and four (28.6%) of the 14 ECOWAS Member States with available data were found to have a readiness level lower than 80% for both WHO-recommended surveillance and diagnostics capacities [28], assuming this was an acceptable threshold (Table S1 in the **Online Supplementary Document**).

### Lack of access to vaccines

Besides surveillance and diagnostics, vaccine access, especially when there is a higher level of population acceptance, remain a critical tool for preventing and controlling infectious disease outbreaks, including mpox [2,4,12]. However, three major weaknesses in the region’s medical countermeasure supply chain and logistics mechanisms could challenge vaccine access in providing adequate population immunity against MPXV, thus, contributing to disparities in morbidity and mortality.

First, there is a lack of local manufacturing capacities for vaccines [28–30]. Currently, 99% of the vaccines used in Africa are produced internationally. This manufacturing is at full capacity and typically involves vaccine substance production (antigen manufacturing) and vaccine product production (formulation/fill/finish). However, the continent’s manufacturing capacity for the remaining 1% is contributed by eight countries and is often limited to formulation/fill/finish [31]. Of these countries, this partial capacity is available in only one ECOWAS Member State – Senegal [31]. This deficiency constrains the ability of many African countries, including the ECOWAS Member States, to access the number of doses of mpox vaccines required for their target populations to enhance community-wide protection [12,14]. This results in the dependency of the Member States on vaccine donations from foreign countries (*e.g.* Spain, Germany, United States). While these are commendable efforts, the unreliable nature of their access mechanism challenges the effective and sustainable implementation of vaccination programmes, increasing the risk of further disease spread and associated health and socioeconomic burden [14–16].

A second challenge is the inadequacy and suboptimal functionality of cold chains [12,32,33]. Ensuring the increased availability of well-functioning cold chains is critical for vaccine access to maintain their potency for real-world vaccine effectiveness. However, this is not yet a reality in the region. If this weakness is not addressed optimally, it may negatively impact the ongoing mpox response. Moreover, several authors have reported on how this deficiency could hamper routine and emergency vaccination efforts, particularly in rural areas and hard-to-reach communities through weak supply chain and logistics infrastructure, inadequate training capacity, limited availability of cold chain equipment, unstable power supply, and poor transport system [12].

The third issues is the non-universality of available mpox vaccines for all ages and high-risk groups [14–16,34]. This weakness is multidimensional and requires careful consideration for vaccine access. First, the mpox vaccine approved for use by the WHO to date is the MVA-BN 2-dose vaccine (JYNNEOS), as evident in most countries with access during the global 2022–23 outbreak [14–16]. However, this is not approved for children under 18 years of age and pregnant women except as ‘off-label’ use, when the benefits of vaccination outweigh the potential risks [34]. More so, MVA-BN makes up most ongoing vaccine donations to African countries. Therefore, its ‘off-label’ use among children under 18 years of age and pregnant women would have to be determined based on the outbreak context and legal landscape in each country. Second, the LC16 single-dose vaccine has been used for children as young as 1–7 years, but it is contraindicated among pregnant women and immunocompromised persons [34]. This suggests that it may also require approvals for use or new clinical trials among children outside Japan, where it was developed and tested.

Further, differences in external vulnerabilities within and between countries in the region, including political and legal landscape, development level, quality of health systems, community geographical terrain, and existing chronic problems, are other underdiscussed problems that could compound surveillance and diagnostics and vaccine access challenges for the mpox response. These could increase the likelihood of missed cases and missed opportunities for vaccination that may cause further spread of the disease and prolong its control efforts [13,17,23,35,36].

These problems are consistent with emerging evidence on logistics (vaccine access) capacities readiness in the region that showed that eight (57.1%) of the 14 ECOWAS Member States studied lacked a readiness level up to an acceptable threshold of 80% (Table S2 in the **Online Supplementary Document**) [28].

### Role of the WAHO and other health actors

The WAHO – a specialised institution of ECOWAS that is responsible for the harmonisation of the policies of its Member States, pooling of resources, and fostering collective and strategic cooperation to ensure attainment of the highest possible standard and protection of the health of the peoples in the region [37], has positioned itself for this mpox outbreak through its Regional Centre for Surveillance and Disease Control. It is currently collaborating with key health actors and stakeholders (*e.g.* Africa CDC, the WHO Regional Office for Africa, the private sector) across all levels of the government and society, recognising the need for an integrated response centred on pragmatism and equity in the ECOWAS region and beyond (p. 8 of the **Online Supplementary Document**).

## PRAGMATISM AND EQUITY CONSIDERATIONS

Pragmatism is understood based on two key ideologies as an epistemological paradigm or as an approach to change [38]. As a paradigm, it recognises the important role of context in shaping the translation of knowledge to policy. This ideology embodies values of pluriversality and contextualisation for local knowledge adaptation. Ultimately, this seeks to establish the practical application of knowledge to improve its ‘usability’ in a real-world situation. As a change, it supports the argument for translating policy to action based on a given context. This ideology represents the values of incrementalism and dynamism for social change, while embracing collective effort in identifying what is feasible under the current circumstances and promoting its implementation to improve the ‘impact’ of knowledge [38].

Similarly, equity is a multidimensional concept involving two major ideologies: equity as a process and as an outcome [39,40]. As a process, equity is defined as the ‘fair treatment, access, opportunity, and advancement for all people, while simultaneously striving to identify and eliminate barriers that prevent the full participation of some’ [39]. As an outcome, it is regarded as the ‘absence of systematic disparities in health (or in the major social determinants of health) between groups with different levels of underlying social advantage/disadvantage – that is, wealth, power, or prestige’ [40] (Box S1 in the **Online Supplementary Document**) [22,38–40].

These principles are strongly advocated by the WHO policy guidance for mpox and other infectious diseases, emphasising equitable access to medical countermeasures among at-risk populations and geographical areas, particularly in low-resource and humanitarian settings [41]. In this context, the WAHO is aligning its public health emergency preparedness and response strategic action plan with the global policy guidelines, including the WHO Mpox Global Strategic Preparedness, Readiness and Response Plan, and the 2024–27 Strategic Framework for Enhancing Prevention and Control of Mpox, to support the West African countries (Box S2 in the **Online Supplementary Document**) [41–43].

## RECOMMENDATIONS

### Short-term overarching strategies and actions

#### Surveillance and diagnostics

To prevent, detect, and respond to the growing burden of this disease effectively and efficiently, priority must be given to three fundamental, interrelated aspects of surveillance and diagnostics, including stigma-sensitive risk communication and community engagement , training of health workers and community volunteers, and decentralised monitoring and testing (**Table 1**) [2,4,25,26,41,42,44–46].

**Table 1:** Short-term strategies and actions for mpox surveillance and diagnostics and vaccine access

Mpox strategy and action	Rationale	Description	Policy implication	Example
Surveillance and diagnostics: stigma-sensitive risk communication and community engagement; training of health workers and community volunteers; decentralised monitoring and testing	Low awareness level or high misinformation rate, stigma related care seeking reluctance, and past outbreaks experience	Targeted messaging: involve multiple sectors, including health, education, and news media; change from ‘fear-based and risk-laden’ to ‘stigma-sensitive and benefit-oriented’ mpox messages; developed to be culturally appropriate using familiar languages with actionable points; presented in audio-visual and print forms; and disseminated by trusted individuals from communities, schools, religious centres, workplaces, MSM/sex workers networks, and health facilities	Increase collective confidence and responsibility to reduce stigmatisation	Public service announcements via radio and television; public awareness and education talks via community campaign, school, religious, workplace, MSM/sex workers network, and health facility activities; electronic messaging via SMS, social media, websites, emails, and other digital platforms; print messaging via billboards and newspapers
	Limited health emergency preparedness and response competencies	Inclusive and competency-based learning: develop locally relevant training contents on mpox surveillance and diagnostics, vaccination, risk communication and community engagement, infection prevention and control, and water, sanitation and hygiene; deliver training leveraging existing training platforms at the national, regional, and global levels	Improve learning and response outcomes	Facilitated learning via physical training centre and/or Zoom; self-directed learning via WhatsApp and/or mobile app and web; simulation-based learning via physical training centre and/or immersive technology, and leverage MoH, Africa CDC IWD, and WHO Academy platforms
	Specific populations, including children and other high-risk groups, are at higher risk and can be targeted at their points of contact with health interventions	Integrated screening, diagnosis, and care: expand access through relevant community-based and health facility-based ‘POA’ sites; mandatory questionnaire-based clinical screening of patients at these sites; the organised transfer of patients with suspected mpox from these sites to ‘POC’ isolation wards for sample collection and care; and the safe transport of the samples to laboratories with PCR/sequencers	Reduce missed cases and improve virus characterisation for optimal control	Community-based POA monitoring and testing via campaign venues, schools, religious centres, workplaces, and MSM/sex workers networks; health facility-based POA via paediatric care, antenatal care, and HIV care clinics; provision of personal protective equipment and laboratory biosafety measures
Vaccine access: phased vaccine rollout; community-based vaccine distribution; dose-sparing vaccine administration	Limited access to vaccines, disproportionate disease risk, and unequal availability of vaccine delivery infrastructure	Risk-based immunisation: rollout for at-risk populations in ‘phase 1’ to stop mpox outbreaks, vulnerable populations in ‘phase 2’ to limit further community spread, underserved communities in ‘phase 3′ to increase population immunity; hub-and-spoke delivery model: distribution from a centralised cold chain to a hub local network with ultra-cold capacity then to spoke local networks with cold storage if health facilities are few, network-cold chain travel distances are long, and outbreak is in the response stage. Point-to-point model: from a centralised cold chain to local networks with cold storage if health facilities are many, network-cold chain travel distances are short, and outbreak is in the recovery stage; low dose prophylaxis: change from ‘two-dose subcutaneous (0.5mL)’ to ‘single-dose intradermal (0.1mL dose)’ for MVA-BN vaccine	Maximising vaccine access and potency for wider community protection	Immunisation via routine and supplementary activities; leverage CBOs and MSM/sex workers networks

CBOs – community-based organisations, CDC – Centres for Disease Control and Prevention, HIV – human immunodeficiency virus, IWD – Institute for Workforce Development, MoH – Ministry of Health, MSM – men who have sex with men, MVA-BN – Modified Vaccinia Ankara–Bavarian Nordic, PCR – polymerase chain reaction, POC – point-of-care, POA – point-of-access, SMS – short message service, WHO – World Health Organization

Implementing these strategies will help ensure that the target high-risk groups are reached while maintaining the continuity of essential health services. Alongside ongoing cross-border surveillance and integrated disease surveillance and response through a One Health lens, this will enhance early care seeking, comprehensive case detection, timely case reporting, and effective linkage to integrated care to reduce the development of new cases and improve outbreak response outcomes. This should further be complemented with vaccine access to accelerate progress towards its control.

#### Vaccine access

With the lack of local vaccine manufacturing capacities, vaccine donation remains a viable option to interrupt transmission and reduce mpox-associated complications and deaths [4,30,31]. To ensure vaccine coverage among target populations and communities while building health system resilience, evidence-based strategies, including phased vaccine rollout, community-based vaccine distribution, and dose-sparing vaccine administration (**Table 1**), must be prioritised [18,34,41,47]. These offer the potential to enable a quicker transition from the early-mid outbreak (response) stage to the late-post outbreak (recovery) stage.

Guided by these short-term pragmatism and equity considerations and their practical applications and metrics based on the WHO ‘5 Cs’ of its health emergency prevention, preparedness, response, and resilience framework (**Table 2**) [34,41,45,47,48], in addition to whole of society-based vulnerability management to improve security in conflict-affected areas, economic stability, social amenities access, health workforce retention, we believe that the global health community will be able to not only control mpox promptly, but also accelerate progress toward its elimination through sustained commitments and investments from countries, regional bodies, and relevant stakeholders.

**Table 2:** Practical application and metrics of short-term pragmatism and equity consideration for mpox

WHO Strategy and Action [41,48]	Pragmatism (in low-resource settings)	Equity (in all settings)
Strengthened collaborative surveillance and detection: strong national integrated disease, threat, and vulnerability surveillance; effective diagnostics and laboratory capacity for pathogen and genomic surveillance; collaborative approaches for event detection, risk assessment, and response monitoring	Increase One Health collaborative surveillance, integrating indicator-based and event-based surveillance approaches in geographical areas with zoonotic transmission to better understand the risk of spillover and spillback events. Implement participatory surveillance for mild to moderate cases through self-reporting leveraging low-cost digital solutions (*e.g.* SMS, WhatsApp, mobile app), ensuring data privacy and security to minimise underascertainment/underreporting bias. Establish decentralised laboratories and explore the use of drones in rural, hard-to-reach, and humanitarian areas without adequate diagnostic capacity for sample transport from the point of collection to reference laboratories to improve case detection with the PCR test and timely reporting.	Foster inclusive, participatory, and respectful collaborations by including diverse high-risk groups: people with lived experiences of mpox, at-risk populations (young children, MSM, health workers, contacts of cases), vulnerable populations (people living with HIV, people on immunosuppressive therapy, pregnant women), and underserved communities (rural, hard-to-reach, humanitarian) in decision-making processes. Disaggregate indicator/event-based surveillance data by all high-risk groups based on the outcomes of interest. Indicator, percentage of: high risk states with integrated surveillance, laboratories with decentralised PCR testing, suspected mpox cases in the high-risk groups tested.
Enhanced community protection: community engagement, risk communication and infodemic management to guide priority actions and strengthen community resilience; population and environmental public health interventions; multisectoral action to respond to community concerns and ensure community welfare	Leverage WhatsApp, social media, and eHealth for targeted, tailored RCCE. Develop vaccination phased rollout and community-based distribution models, integrated into existing health services (*e.g.* HIV, routine/supplementary immunisation), [41,47] including psychosocial support via local networks. For the MVA-BN vaccine, use a single-dose intradermal administration (0.1 mL dose) of vaccine as a dose-sparing option instead of the conventional two-dose subcutaneous administration (0.5 mL) to improve coverage [34].	Ensure age-appropriate, stigma-sensitive RCCE. Provide vaccination and therapeutics and other public health and social interventions in high-risk communities with active outbreaks and larger proportions of their total populations with high-risk groups, as informed by local evidence and targets. Indicator, percentage of: communities with mpox RCCE, high-risk communities with integrated vaccination, high-risk communities with vaccination linked to psychosocial support.
Safe and scalable care: scalable clinical care during emergencies; protection of health workers and patients; maintenance of essential health services	Leverage trained community health workers and volunteers for contact tracing, isolation, vaccination, and clinical care in hospitals or homes, or local networks. Intensify hospital and community IPC and WASH interventions and trainings, including provision of PPE.	Ensure provision of standardised training on contact tracing, isolation, vaccination, clinical care, IPC, WASH, and PPE use and disposal for health workers/volunteers. Deliver age-appropriate clinical care for high-risk groups based on local evidence and protocols. Indicator, percentage of: hospitals and communities with IPC, WASH, and PPE.
Equitable access to medical countermeasures: fast-track research and development; scalable manufacturing; end-to-end health emergency supply chains	Use a regional or continental resource pooling strategy to improve equitable access to MCM. Establish community coalition/advocacy groups for research, and vaccine access *via* vaccine donation, development of solar-powered cold chain, and manufacturing technology transfer.	Implement a risk-based strategy for the allocation of MCM to high-risk areas and their high-risk groups. Ensure a high-risk group-led coalition/ advocacy. Indicator, percentage of: states with risk-based MCM allocation.
Emergency coordination: strengthened workforce capacities for health emergencies; health emergency preparedness, readiness and resilience; health emergency alert and response coordination	Improve health workers/volunteers’ knowledge and skills on mpox leveraging new or existing online health emergency communities of practice using WhatsApp and Zoom [45]. Strengthen new or existing health EC and IMS systems with digital solutions.	Provide contextualised, standardised mpox trainings on EC, IMS, and leadership to the health workers/volunteers. Improve access of the EC/IMS and the workforce to internet/other digital solutions. Indicator, percentage of: states with digitally-supported EC and IMS systems.

EC – emergency coordination, IMS – incident management support, IPC – infection prevention and control, MCM – men who have sex with men, PCR – polymerase chain reaction, PPE – personal protective equipment, RCCE – risk communication and community engagement, WASH – water, sanitation, and hygiene

### Long-term overarching strategies and actions

To maximise the benefits of these short-term considerations, they must be treated as mutually non-exclusive to the long-standing, deep-rooted systemic issues in the global health architecture and health systems. The well-documented barriers in the current state of the global health architecture, including systems of oppression (colonialism and racism) and power and privilege imbalance (patriarchy and lack of diversity, inclusion, and equity), need to be carefully addressed, as they pose the greatest threat to universal health coverage, health security, health equity, and sustainable development goals [49–52].

The increasing impact of climate change and emerging political polarisation worldwide, coupled with the rapidly changing global health funding landscape, threaten priority setting and call for moving advocacy into tangible actions [53–55], indicating that innovative solutions are urgently needed to reform the global health architecture on a path of sustainability. This is crucial for responding effectively, efficiently, and equitably to mpox and other public health emergencies. A good example of achieving this would be by reforming the global health architecture through systems delineation and processes changes, strengthening regional health systems through positions and contributions critiquing and self-reliant financing, and increasing investments for primary and secondary pandemic prevention through One Health ecological surveillance and collaborative surveillance (p. 11–17 and Box S3 in the **Online Supplementary Document** [11,56–76]).

### IMPLEMENTATION PRIORITIES

Even if these recommendations are considered, the intended response outcomes might not be achieved without a roadmap that highlights the priorities for their implementation. Examples of such priorities include a clear coordination mechanism, cohesive capacity building, consistent risk assessment, unified case reporting, locally led and internationally supported research, commodity and vaccine security, and integrated monitoring and evaluation (p. 20, Box S4, and Table S3 in the **Online Supplementary Document**) [22,38,41,43,77–85].

## CONCLUSION

Insights from this viewpoint suggest high mpox transmission potential in the endemic part of West Africa and possible outbreak expansion to bordered and non-endemic countries in Africa and beyond, given suboptimal surveillance and diagnostics and vaccine access readiness capacities. This current epidemiological context highlights the need for strengthening mpox response capacities in the affected and unaffected parts of the region, while prioritising resources for the most at-risk countries to increase health workers training, event-based surveillance, triple packing sample transport materials, laboratory biosafety measures, and cold chains for improved health outcomes.

## Additional material


Online Supplementary Document

## References

[1] Mitjà O Ogoina D Titanji BK Galvan C Muyembe JJ Marks M Monkeypox. Lancet. 2023;401:60-74. 10.1016/S0140-6736(22)02075-X 36403582 PMC9671644

[2] Laurenson-Schafer H Sklenovská N Hoxha A Kerr SM Ndumbi P Fitzner J Description of the first global outbreak of mpox: an analysis of global surveillance data. Lancet Glob Health. 2023;11:e1012–23. 10.1016/S2214-109X(23)00198-5 37349031 PMC10281644

[3] United States Centers for Disease Control and Prevention. 2022–2023 Mpox Outbreak Global Map. Available: https://archive.cdc.gov/#/details?url=https://www.cdc.gov/poxvirus/mpox/response/2022/world-map.html. Accessed: 18 August 2024.

[4] Ndembi N Folayan MO Ngongo N Ntoumi F Ogoina D El Rabbat M Mpox outbreaks in Africa constitute a public health emergency of continental security. Lancet Glob Health. 2024;12:e1577–9. 10.1016/S2214-109X(24)00363-2 39178878

[5] World Health Organization. WHO Director-General declares mpox outbreak a public health emergency of international concern. 14 August 2024. Available: https://www.who.int/news/item/14-08-2024-who-director-general-declares-mpox-outbreak-a-public-health-emergency-of-international-concern. Accessed: 18 August 2024.

[6] Africa Centres for Disease Control and Prevention. Africa CDC briefing on mpox outbreak in the continent | Sept. 6, 2024 [video]. 6 September 2024. Available: https://www.youtube.com/watch?v=pa18cfknrWA. Accessed: 7 September 2024.

[7] Africa Centres for Disease Control and Prevention. Outbreak Report, 26 August 2024: Mpox Situation in Africa. 26 August 2024. Available: https://africacdc.org/download/outbreak-report-26-august-2024-mpox-situation-in-africa/. Accessed: 27 August 2024.

[8] World Health Organization. Mpox: key facts. 26 August 2024. Available: https://www.who.int/news-room/fact-sheets/detail/mpox. Accessed: 27 August 2024.

[9] World Health Organization. Multi-country monkeypox outbreak in non-endemic countries: update. 21 May 2022. Available: https://www.who.int/emergencies/disease-outbreak-news/item/2022-DON385. Accessed: 26 August 2024.

[10] Rivers C Watson C Phelan AL The Resurgence of Mpox in Africa. JAMA. 2024;332:1045–6. 10.1001/jama.2024.17829 39163302

[11] Adetifa I Muyembe JJ Bausch DG Heymann DL Mpox neglect and the smallpox niche: a problem for Africa, a problem for the world. Lancet. 2023;401:1822–4. 10.1016/S0140-6736(23)00588-3 37146622 PMC10154003

[12] Moyo E Musuka G Murewanhema G Moyo P Dzinamarira T Monkeypox outbreak: a perspective on Africa’s diagnostic and containment capacity. Int J Infect Dis. 2022;123:127–30. 10.1016/j.ijid.2022.08.016 36007687 PMC9534167

[13] Reynolds MG Doty JB McCollum AM Olson VA Nakazawa Y Monkeypox re-emergence in Africa: a call to expand the concept and practice of One Health. Expert Rev Anti Infect Ther. 2019;17:129-39. 10.1080/14787210.2019.1567330 30625020 PMC6438170

[14] Africa Centres for Disease Control and Prevention. Africa CDC and Bavarian Nordic partner to boost mpox vaccine production in Africa. 17 August 2024. Available: https://africacdc.org/news-item/africa-cdc-and-bavarian-nordic-partner-to-boost-mpox-vaccine-production-in-africa/. Accessed: 26 August 2024.

[15] Adepoju P Mpox declared a public health emergency. Lancet. 2024;404:e1–2. 10.1016/S0140-6736(24)01751-3 39182498

[16] Taylor L First mpox vaccines arrive in Africa as officials work “blindly” to contain outbreaks. BMJ. 2024;386:q1897. 10.1136/bmj.q1897 39209462

[17] Chikezie NC Shomuyiwa DO Okoli EA Onah IM Adekoya OO Owhor GA Addressing the issue of a depleting health workforce in sub-Saharan Africa. Lancet. 2023;401:1649–50. 10.1016/S0140-6736(23)00720-1 37210110

[18] United Nations Children’s Fund. Multiple and simultaneous epidemics on the rise in West and Central Africa. 16 November 2022. Available: https://www.unicef.org/wca/press-releases/multiple-and-simultaneous-epidemics-rise-west-and-central-africa. Accessed: 26 August 2024.

[19] Gao S Zeng Z Zhai Y Chen F Feng X Xu H Driving effect of multiplex factors on Mpox in global high-risk region, implication for Mpox based on one health concept. One Health. 2023;17:100597. 10.1016/j.onehlt.2023.100597 38024251 PMC10665165

[20] Rutter H Savona N Glonti K Bibby J Cummins S Finegood DT The need for a complex systems model of evidence for public health. Lancet. 2017;390:2602–4. 10.1016/S0140-6736(17)31267-9 28622953

[21] Sheikh K Peters D Agyepong IA Abimbola S Ghaffar A Swaminathan S Learning is a means to progress and empowerment for health systems. BMJ Glob Health. 2022;7 Suppl 7:e010572. 10.1136/bmjgh-2022-010572 36130795 PMC9490602

[22] World Health Organization. Strategic toolkit for assessing risk: a comprehensive toolkit for all-hazards health emergency risk assessment. Geneva, Switzerland: World Health Organization; 2021. Available: https://www.who.int/publications/i/item/9789240036086. Accessed: 26 August 2024.

[23] World Health Organization. Mpox – African region: WHO risk assessment. 22 August 2024. Available: https://www.who.int/emergencies/disease-outbreak-news/item/2024-DON528. Accessed: 26 August 2024.

[24] West African Health Organization. Confirmed Mpox cases in the ECOWAS region for 2024 [not available online]. 3 September 2024.

[25] Sam-Agudu NA Martyn-Dickens C Ewa AU A global update of mpox (monkeypox) in children. Curr Opin Pediatr. 2023;35:193–200. 10.1097/MOP.0000000000001232 36809304

[26] Pronyk PM de Alwis R Rockett R Basile K Boucher YF Pang V Advancing pathogen genomics in resource-limited settings. Cell Genom. 2023;3:100443. 10.1016/j.xgen.2023.100443 38116115 PMC10726422

[27] World Health Organization Regional Office for Africa. Bolstering monkeypox laboratory testing in Africa. 30 June 2022. Available: https://www.afro.who.int/news/bolstering-monkeypox-laboratory-testing-africa. Accessed: 26 August 2024.

[28] Lokossou VK Awori AS Fatimehin V Usman AB Sogbossi L Agbla F Assessing mpox epidemic readiness status in ECOWAS region: strengths, gaps, and recommendations for an improved response. Pan Afr Med J. 2025;50:2. 10.11604/pamj.supp.2025.50.1.46077 PMC12594583 41209597

[29] Ekström AM Tomson G Wanyenze RK Bhutta ZA Kyobutungi C Binagwaho A Addressing production gaps for vaccines in African countries. Bull World Health Organ. 2021;99:910–2. 10.2471/BLT.21.287381 34866689 PMC8640685

[30] Ogunkola IO Abiodun OE Bale BI Elebesunu EE Ujam SB Umeh IC Monkeypox vaccination in the global south: Fighting a war without a weapon. Clin Epidemiol Glob Health. 2023;22:101313. 10.1016/j.cegh.2023.101313 37220529 PMC10195808

[31] Wellcome Trust. Scaling up African vaccine manufacturing capacity: perspectives from the African vaccine-manufacturing industry. London, UK: Wellcome Trust; 2023. Available: https://wellcome.org/reports/scaling-african-vaccine-manufacturing-capacity. Accessed: 27 August 2024.

[32] Babatunde OA Olatunji MB Omotajo OR Ikwunne OI Hamzat Z Sola ST A comparative assessment of cold chain management using the outbreak of circulating vaccine-derived polio virus type 2 as a surrogate marker in Oyo State, Nigeria-2019. Pan Afr Med J. 2020;37:313. 10.11604/pamj.2020.37.313.26152 33654532 PMC7896533

[33] Ashok A Brison M LeTallec Y Improving cold chain systems: Challenges and solutions. Vaccine. 2017;35:2217–23. 10.1016/j.vaccine.2016.08.045 27670076

[34] World Health Organization. Smallpox and mpox (orthopoxviruses): WHO position paper, August 2024. Geneva, Switzerland: World Health Organization; 2024. Available: https://www.who.int/publications/i/item/who-wer-9934-429-456. Accessed: 26 August 2024.

[35] Lokossou VK Usman AB Sombie I Paraiso MN Balogun MS Umeokonkwo CD COVID-19 pandemic in Economic Community of West African States (ECOWAS) region: implication for capacity strengthening at Point of Entry. Pan Afr Med J. 2021;39:67. 10.11604/pamj.2021.39.67.29089 34422190 PMC8363963

[36] Izugbara C Bakare S Sebany M Ushie B Wekesah F Njagi J Regional legal and policy instruments for addressing LGBT exclusion in Africa. Sex Reprod Health Matters. 2020;28:1–14. 10.1080/26410397.2019.1698905 31928329 PMC7887941

[37] West African Health Organization. Who we are. Available: https://www.wahooas.org/web-ooas-prod/en/who-we-are. Accessed: 26 August 2024.

[38] Smith J Boro E Kwong EJL Schmidt-Sane M Resisting unchecked pragmatism in global health. Lancet Glob Health. 2023;11:e1176–7. 10.1016/S2214-109X(23)00208-5 37329894

[39] Network of Schools of Public Policy, Affairs, and Administration. Taskforce on diversity, inclusion, and equity: toward a comprehensive framework and action plan September 2020. October 2020. Available: https://www.naspaa.org/sites/default/files/docs/2020-12/21%20DEI%20Task%20Force%20Final%20Report%2010-5-20.pdf. Accessed: 26 August 2024.

[40] Braveman P Gruskin S Defining equity in health. J Epidemiol Community Health. 2003;57:254–8. 10.1136/jech.57.4.254 12646539 PMC1732430

[41] World Health Organization. Mpox global strategic preparedness and response plan. Geneva, Switzerland: World Health Organization; 2024. Available: https://www.who.int/publications/m/item/mpox-global-strategic-preparedness-and-response-plan. Accessed: 26 August 2024.

[42] World Health Organization. Strategic framework for enhancing prevention and control of mpox 2024–2027. Geneva, Switzerland: World Health Organization. Available: https://www.who.int/publications/i/item/9789240092907. Accessed: 26 August 2024.

[43] West African Health Organization. Emergency meeting on mpox: press release. Available: https://www.wahooas.org/web-ooas/en/actualites/emergency-meeting-mpox-press-release. Accessed: 26 August 2024.

[44] Ogunyemi KO Lokossou VK Sogbossi LS Ohuabunwo C Antara S Kebede S A pragmatic assessment of contextual fit and feasibility for e-learning implementation among public health workers in 16 West African countries: a mixed-methods study. J Glob Health Econ Policy. 2024;4:e2024. 10.52872/001c.123616

[45] Kessy SJ Gon G Alimi Y Bakare WA Gallagher K Hornsey E Training a Continent: A Process Evaluation of Virtual Training on Infection Prevention and Control in Africa During COVID-19. Glob Health Sci Pract. 2023;11:e2200051. 10.9745/GHSP-D-22-00051 37116932 PMC10141425

[46] Boodman C Heymann DL Peeling RW Inadequate diagnostic capacity for monkeypox-sleeping through the alarm again. Lancet Infect Dis. 2023;23:140–1. 10.1016/S1473-3099(22)00744-7 36402145 PMC9671516

[47] Xu X Rodgers MD Guo WG A hub-and-spoke design for ultra-cold COVID-19 vaccine distribution. Vaccine. 2021;39:6127–36. 10.1016/j.vaccine.2021.08.069 34509324 PMC8384589

[48] World Health Organization. Strengthening health emergency prevention, preparedness, response and resilience. Geneva, Switzerland: World Health Organization; 2023. Available: https://cdn.who.int/media/docs/default-source/emergency-preparedness/who_hepr_wha2023-21051248b.pdf?sfvrsn=a82abdf4_3&download=true. Accessed: 26 August 2024.

[49] Gostin LO Friedman EA Finch A The global health architecture: governance and international institutions to advance population health worldwide. Milbank Q. 2023;101 S1:734–69. 10.1111/1468-0009.12627 37096621 PMC10126971

[50] McCoy D Kapilashrami A Kumar R Rhule E Khosla R Developing an agenda for the decolonization of global health. Bull World Health Organ. 2024;102:130–6. 10.2471/BLT.23.289949 38313156 PMC10835633

[51] Abimbola S Asthana S Montenegro C Guinto RR Jumbam DT Louskieter L Addressing power asymmetries in global health: imperatives in the wake of the COVID-19 pandemic. PLoS Med. 2021;18:e1003604. 10.1371/journal.pmed.1003604 33886540 PMC8101997

[52] Büyüm AM Kenney C Koris A Mkumba L Raveendran Y Decolonising global health: if not now, when? BMJ Glob Health. 2020;5:e003394. 10.1136/bmjgh-2020-003394 32759186 PMC7409954

[53] Romanello M Napoli CD Green C Kennard H Lampard P Scamman D The 2023 report of the Lancet Countdown on health and climate change: the imperative for a health-centred response in a world facing irreversible harms. Lancet. 2023;402:2346–94. 10.1016/S0140-6736(23)01859-7 37977174 PMC7616810

[54] Wong BLH Nordström A Piot P Clark H Global Health Diplomacy Partners From polycrisis to metacrisis: harnessing windows of opportunity for renewed political leadership in global health diplomacy. BMJ Glob Health. 2024;9:e015340. 10.1136/bmjgh-2024-015340 38637121 PMC11029238

[55] World Health Organization. How WHO is funded. 2025. Available: https://www.who.int/about/funding. Accessed: 3 January 2025.

[56] Rifkin SB Alma Ata after 40 years: Primary Health Care and Health for All-from consensus to complexity. BMJ Glob Health. 2018;3 Suppl 3:e001188. 10.1136/bmjgh-2018-001188 30622747 PMC6307566

[57] Lomazzi M Borisch B Laaser U The Millennium Development Goals: experiences, achievements and what’s next. Glob Health Action. 2014;7:23695. 10.3402/gha.v7.23695 24560268 PMC3926985

[58] Liu Y Du J Wang Y Cui X Dong J Gu P Overlooked uneven progress across sustainable development goals at the global scale: Challenges and opportunities. Innovation (Camb). 2024;5:100573. 10.1016/j.xinn.2024.100573 38379792 PMC10876912

[59] Spicer N Agyepong I Ottersen T Jahn A Ooms G ‘It’s far too complicated’: why fragmentation persists in global health. Global Health. 2020;16:60. 10.1186/s12992-020-00592-1 32646471 PMC7344046

[60] Pushkaran A Chattu VK Narayanan P A critical analysis of COVAX alliance and corresponding global health governance and policy issues: a scoping review. BMJ Glob Health. 2023;8:e012168. 10.1136/bmjgh-2023-012168 37793808 PMC10551961

[61] Usher AD A beautiful idea: how COVAX has fallen short. Lancet. 2021;397:2322–5. 10.1016/S0140-6736(21)01367-2 34147145 PMC8494620

[62] Boyce MR Sorrell EM Standley CJ An early analysis of the World Bank’s Pandemic Fund: a new fund for pandemic prevention, preparedness and response. BMJ Glob Health. 2023;8:e011172. 10.1136/bmjgh-2022-011172 36599499 PMC9815014

[63] Ndembi N Dereje N Nonvignon J Aragaw M Raji T Fallah MP Financing pandemic prevention, preparedness and response: lessons learned and perspectives for future. Global Health. 2024;20:65. 10.1186/s12992-024-01066-4 39169389 PMC11337782

[64] Torres Munguía JA Badarau FC Díaz Pavez LR Martínez-Zarzoso I Wacker KM A global dataset of pandemic- and epidemic-prone disease outbreaks. Sci Data. 2022;9:683. 10.1038/s41597-022-01797-2 36357405 PMC9648436

[65] Chafee Z Coming into Equity with Clean Hands. II. Mich Law Rev. 1949;47:1065–96. 10.2307/1284236

[66] Nonvignon J Soucat A Ofori-Adu P Adeyi O Making development assistance work for Africa: from aid-dependent disease control to the new public health order. Health Policy Plan. 2024;39 Supplement_1:i79-92. 10.1093/heapol/czad092 38253444 PMC10803194

[67] World Health Organization. WHO African region health expenditure atlas 2023. Geneva, Switzerland: World Health Organization; 2023. Available: https://www.afro.who.int/publications/who-african-region-health-expenditure-atlas-2023-0. Accessed: 22 October 2024.

[68] Apeagyei AE Lidral-Porter B Patel N Solorio J Tsakalos G Wang Y Financing health in sub-Saharan Africa 1990-2050: Donor dependence and expected domestic health spending. PLOS Glob Public Health. 2024;4:e0003433. 10.1371/journal.pgph.0003433 39196881 PMC11355530

[69] PSC Report. Financial independence is key to stronger AU partnerships. Institute for Security Studies. 13 February 2023. Available: https://issafrica.org/iss-today/financial-independence-is-key-to-stronger-au-partnerships. Accessed: 22 October 2024.

[70] Stapel S Söderbaum F European foreign aid to regional organisations in Africa: bullies, overseers, micromanagers and samaritans. Third World Q. 2023;44:1699–717. 10.1080/01436597.2023.2202848

[71] Reeves A Gourtsoyannis Y Basu S McCoy D McKee M Stuckler D Financing universal health coverage–effects of alternative tax structures on public health systems: cross-national modelling in 89 low-income and middle-income countries. Lancet. 2015;386:274–80. 10.1016/S0140-6736(15)60574-8 25982041 PMC4513966

[72] Fanelli S Salvatore FP De Pascale G Faccilongo N Insights for the future of health system partnerships in low- and middle-income countries: a systematic literature review. BMC Health Serv Res. 2020;20:571. 10.1186/s12913-020-05435-8 32571317 PMC7310020

[73] Alilio M Hariharan N Lugten E Garrison K Bright R Owembabazi W Strategies to Promote Health System Strengthening and Global Health Security at the Subnational Level in a World Changed by COVID-19. Glob Health Sci Pract. 2022;10:e2100478. 10.9745/GHSP-D-21-00478 35487550 PMC9053150

[74] World Health Organization Regional Office for Africa. Supply bottleneck, financial challenges fuel delays in Africa’s COVID-19 vaccine rollout. 20 May 2021. Available: https://www.afro.who.int/news/supply-bottleneck-financial-challenges-fuel-delays-africas-covid-19-vaccine-rollout. Accessed: 22 Oct 2024.

[75] Plowright RK Ahmed AN Coulson T Crowther TW Ejotre I Faust CL Ecological countermeasures to prevent pathogen spillover and subsequent pandemics. Nat Commun. 2024;15:2577. 10.1038/s41467-024-46151-9 38531842 PMC10965931

[76] Gessain A Nakoune E Yazdanpanah Y Mbala-Kingebeni P Ntoumi F Nakouné E Monkeypox. N Engl J Med. 2022;387:1783–93. 10.1056/NEJMra2208860 36286263

[77] Nachega JB Sam-Agudu NA Ogoina D Mbala-Kingebeni P Ntoumi F Nakouné E The surge of mpox in Africa: a call for action. Lancet Glob Health. 2024;12:e1086–8. 10.1016/S2214-109X(24)00187-6 38735300

[78] Africa Centres for Disease Control and Prevention. Mpox continental preparedness and response plan for Africa. Addis Ababa, Ethiopia: Africa Centres for Disease Control and Prevention; 2024. Available: https://africacdc.org/download/mpox-continental-preparedness-and-response-plan-for-africa/. Accessed: 6 September 2024.

[79] Damschroder LJ Reardon CM Widerquist MAO Lowery J The updated Consolidated Framework for Implementation Research based on user feedback. Implement Sci. 2022;17:75. 10.1186/s13012-022-01245-0 36309746 PMC9617234

[80] World Health Organization. Nine strategies for developing a scaling-up strategy. Geneva, Switzerland: World Health Organization; 2010. Available: https://www.who.int/publications/i/item/9789241500319. Accessed: 7 September 2024.

[81] Ogunyemi KO McNabb S Lokossou V Sogbossi LS Nyenswah T Ohuabunwo C Developing a new pragmatic tool for assessing contextual fit and feasibility of evidence-based interventions towards effective implementation in global health. BMJ Glob Health. 2025;10:e015931. 10.1136/bmjgh-2024-015931 40240054 PMC12184394

[82] Proctor E Silmere H Raghavan R Hovmand P Aarons G Bunger A Outcomes for implementation research: conceptual distinctions, measurement challenges, and research agenda. Adm Policy Ment Health. 2011;38:65–76. 10.1007/s10488-010-0319-7 20957426 PMC3068522

[83] Glasgow RE Harden SM Gaglio B Rabin B Smith ML Porter GC RE-AIM Planning and Evaluation Framework: Adapting to New Science and Practice With a 20-Year Review. Front Public Health. 2019;7:64. 10.3389/fpubh.2019.00064 30984733 PMC6450067

[84] Chambers DA Norton WE The adaptome: advancing the science of intervention adaptation. Am J Prev Med. 2016;51(4 Suppl 2):S124–31. 10.1016/j.amepre.2016.05.011 27371105 PMC5030159

[85] Bochner AF Makumbi I Aderinola O Abayneh A Jetoh R Yemanaberhan RL Implementation of the 7-1-7 target for detection, notification, and response to public health threats in five countries: a retrospective, observational study. Lancet Glob Health. 2023;11:e871–9. 10.1016/S2214-109X(23)00133-X 37060911 PMC10156425

