# Supplementary material for: Strengthening the mpox response: how do we balance pragmatism and equity in resource-constrained settings?
Source: J Glob Health. 2025 Nov 14;15:03036. doi: 10.7189/jogh.15.03036 (PMC12615003; doi:10.7189/jogh.15.03036)
Supplement: Online Supplementary Document [file jogh-15-03036-s001.pdf]

Supplement to: Lokossou VK, Ogunyemi KO, Usman AB, Kamdem S, Ahanhanzo CD, Agbla F, Aïssi MA, Sombié I. Strengthening the mpox response: how do we balance pragmatism and equity in resource-constrained settings? J Glob Health. 2025;15:03036.

Framework for Analysis

Recognizing the critical role of learning as a means to transforming health systems, the analysis underlying insights provided in this paper was based on the Learning Health System framework [1-2]. This framework is considered appropriate, as it supports translation of evidence on complex problems (e.g., mpox, pragmatism, equity) within a complex system into practice. This was achieved through the triangulation of knowledge from government/institutional reports (surveillance) and literature *information*, physical and online *deliberation*, and *action and praxis* from 20 years of collective, multisectoral public health emergency response experience in a low-resource setting [1].

Most of the data underlying this analysis were selected based on three criteria: 1) relevance, 2) reliability, and 3) real-world value.

Relevance: government/institutional and literature materials were considered relevant if they provided data on mpox from the African perspective to balance the need for contextualized knowledge. Examples included reports from the West African Health Organization (WAHO), the Africa Centres for Disease Control and Prevention (Africa CDC), and the World Health Organization Regional Office for Africa (WHO AFRO), as well as African authors-led journal articles, if available.

Reliability: it was assumed that data from these sources would have undergone rigorous verification and peer review processes to minimize potential information biases.

Real-world value: these data sources were considered eligible based on the assumption that our primary target audiences such as African Health Ministers, healthcare and public health leaders, other health actors, non-health stakeholders, and international development partners, would be adapting their national/subnational and institutional response strategy and action plans in alignment with those from these lead regional and continental health institutions.

Significance

Through spillovers from animals to humans (zoonotic transmission) and sustained human-to-human transmission via close physical contact with infected persons, contaminated materials, or infected animals, MPVX has been implicated in several outbreaks in Africa and globally, claiming many lives [3-4]. Following its discovery in 1958 among monkeys, the first human case was reported in 1970, leading to major outbreaks in endemic countries, particularly in the Democratic Republic of the Congo and Nigeria [3]. There have also been reports of mpox cases in non-endemic countries (e.g., the United Kingdom, Singapore) [3].

More than 50 years after the discovery of mpox, its epidemiological, virological, and clinical patterns have evolved substantially. When its past outbreaks were compared with the 2022-2023 outbreak, its distribution was observed to change from young children in endemic countries to men who have sex with men (MSM) in endemic and non-endemic countries [3-5]. Equally, the scale of its transmission that previously resulted in sporadic-episodic outbreaks is now large and often involving multiple countries [3-5]. These changes have made its control and elimination very challenging. Like other disease outbreaks, the complex and dynamic interactions between numerous factors involving an infectious agent (e.g., transmissibility, severity, adaptation), a host (sociodemographics, population immunity, population behaviour), and the environment (population and social structure, health system, climate change) are thought to be responsible for this problem [3,6].

Specifically, decades-long periods of declining population immunity from smallpox vaccination cross-protection and changes in populations structure following its eradication in 1980, low political commitments and financing, suboptimal surveillance and diagnostics, and underinvestment in research and development, in part caused by the existing global health architecture that is inequitable and unjust, have been attributed to the worsening state of mpox [7-9]. Further making infectious diseases like mpox hard to control is the recurrence of conflict and resultant political instability and insecurity, which have led to some parts of West Africa lacking adequate and sustained public health emergency management [10].

Regrettably, Africa faces a significant challenge in responding to the mpox due to scarce resources [7-9]. The region struggles with suboptimal access to medical countermeasures (MCM), including vaccines, therapeutics, and diagnostics, largely because of a lack of local manufacturing capacities and weak supply chain and logistics mechanisms. In addressing this deficiency, the Africa CDC has taken steps to secure more than 200,000 doses of mpox vaccine (of the 10 million doses pledged) as of early September 2024 and is still expecting more donations for the whole continent. Of these, 175,420 doses are expected from the European Commission's Health Emergency Preparedness and Response (HERA) and 40,000 doses from Bavarian Nordic, amongst other vaccine promises [11-13].

Despite these acts of solidarity, it is still unclear how the vaccine gap (vaccine doses for 80% of the target population) has been reliably estimated to secure the needed vaccines to interrupt transmission in the active phase of the outbreak and achieve herd immunity in the longer term. To have a reliable estimate of this gap, access to recent and complete data on all at-risk, target populations (e.g., young children, MSM) is crucial, and this may be challenged due to weak vital statistics and stigmatization concerns for MSM. Additionally, achieving a timely closure of this gap may be almost impossible, as the availability of self-sufficient vaccine manufacturing factories in the region would require multiple years of efforts. Further compounding this issue is the shortage of health workers, the burden of multiple and concurrent disease outbreaks, and poor research infrastructure [7,14-15].

To control this ongoing outbreak efficiently, pragmatism and equity must be the core principles that drive its political and response actions in the short and long terms. By leveraging existing health system capacities (e.g., disease surveillance system, One Health network); social systems (community of practice, civil society); and digital technology (social media, eHealth), this ongoing outbreak presents a unique opportunity for national, regional, and global health actors to collaborate with the communities, private sector, academia, and relevant stakeholders in mounting a well-coordinated response, in line with the International Health Regulations (IHR 2005) and global best practices.

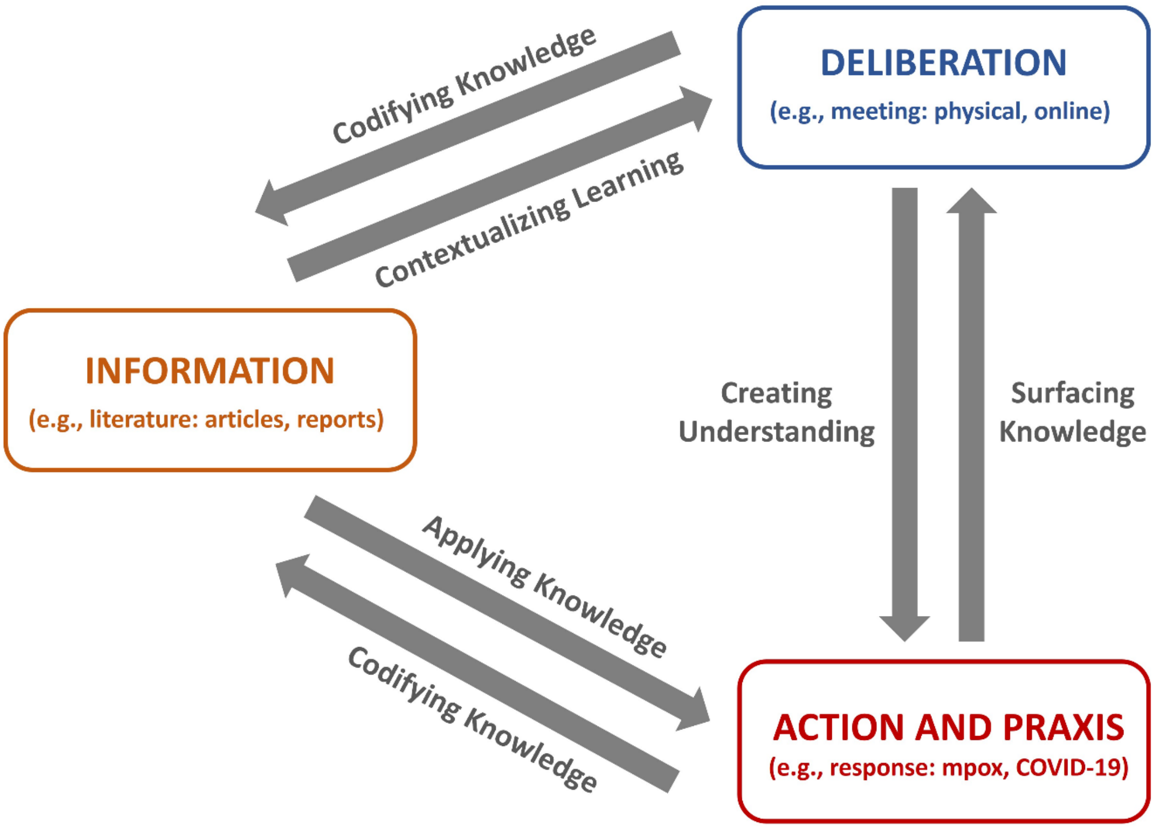

Figure S1. Information-Deliberation-Action and Praxis relationship of the Learning Health Systems Framework that guided the formal analysis of data used in generating insights in this study on mpox response [1].

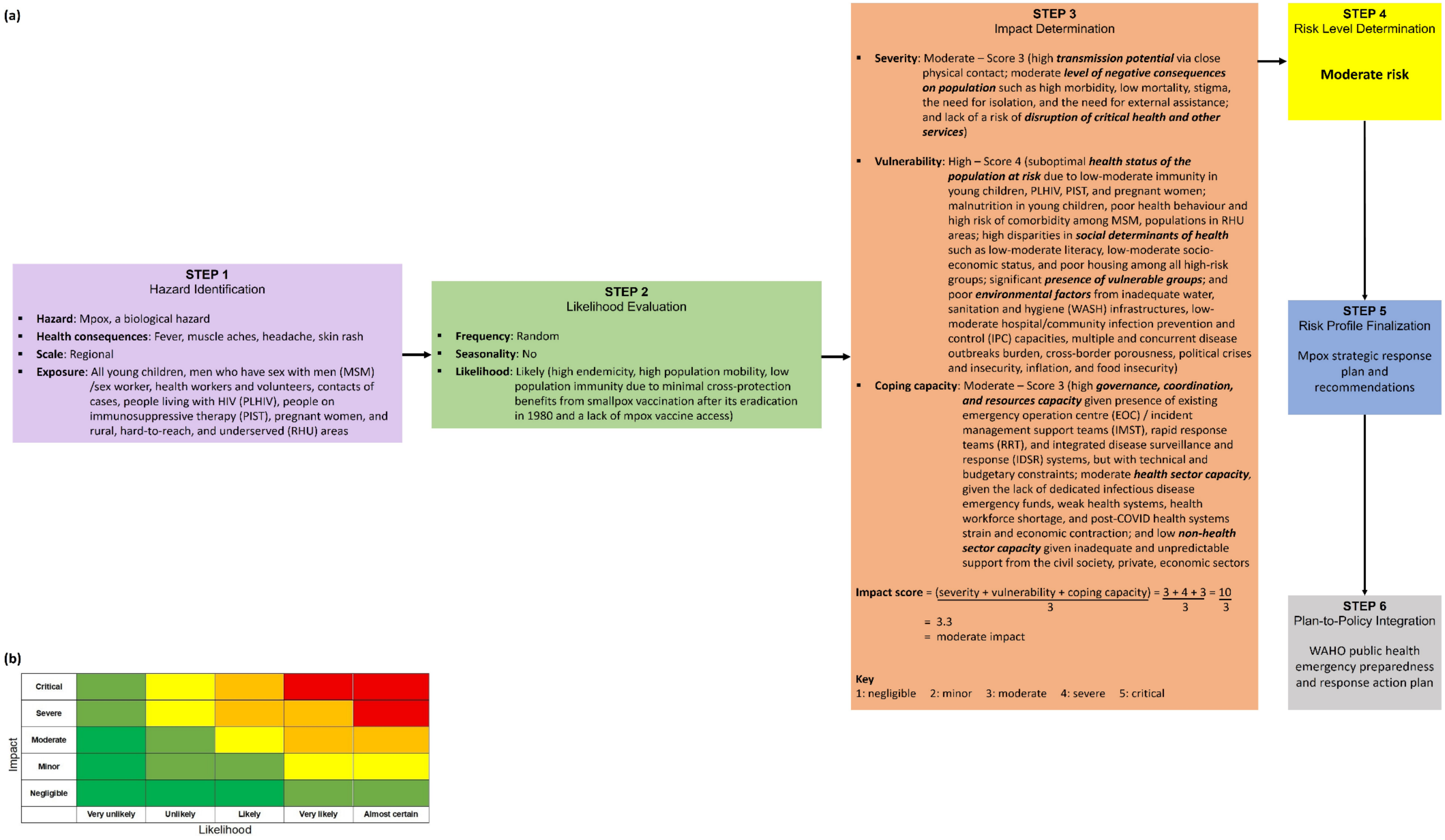

Figure S2. Rapid assessment of mpox risk in West Africa, as of September 02, 2024 using the World Health Organization (WHO) Strategic Toolkit for Assessing Risks (STAR) tool. Figure (**a**) shows the steps for conducting the risk assessment. Figure (**b**) shows the 5x5 matrix for visualizing the risk of mpox outbreak expansion based on its likelihood rating in step 2 and impact score in step 3, where deep green: very low, light green: low, yellow: moderate, orange: high, and red: very high [16].

Table S1. West African countries with suboptimal surveillance and diagnostic capacity readiness level based on an 80% acceptable threshold [17].

Country	Surveillance Readiness Level (%)
Cabo Verde	50
Côte d'Ivoire[†]	50
Gambia	70
Ghana	70
Guinea	50
Guinea-Bissau	**30**
Mali	70
Nigeria[†]	50
Sierra Leone	50
Togo	70
	**Diagnostics Readiness Level (%)**
Cabo Verde	75
Côte d'Ivoire	75
Guinea-Bissau	**63**
Mali	75

The West African countries surveyed included the above highlighted 10 countries and 4 additional countries: Benin, Burkina Faso, Liberia, and Senegal. Note: Countries are arranged in alphabetical order. Bold figure – indicates the lowest capacity. † – mpox-affected countries.

Interpretation – Of the three mpox-affected West African countries including Liberia, this suggests poor surveillance readiness capacity in two (66.7%) countries, Côte d'Ivoire and Nigeria, with both reporting a level of 50% each, and a weak diagnostics capacity in one (33.3%) country, Côte d'Ivoire. Further, it was found that half of the countries did not have a sufficiently trained health workforce on mpox surveillance, with the lowest capacity reported in Liberia, Guinea-Bissau, and Côte d'Ivoire. It was also reported that two countries, Côte d'Ivoire and Cabo Verde, lacked an event-based surveillance system. In addition, while PCR tests and reagents were readily available for diagnosis in all countries, genomic sequencing remained suboptimal. The same study also showed that 10 (71.4%) countries had access to triple packing sample transport materials, with constraints reported in Guinea-Bissau, Cabo Verde, Gambia, and Côte d'Ivoire. Lastly, adequate laboratory biosafety measures, including an autoclave, were also reported to be lacking in only Guinea-Bissau.

Table S2. West African countries with suboptimal logistics (vaccine access) capacity readiness level based on an 80% acceptable threshold [17].

Country	Logistics (Vaccine Access) Readiness Level (%)
Burkina Faso	62
Cabo Verde	**38**
Côte d'Ivoire†	77
Guinea	**77**
Guinea-Bissau	69
Mali	77
Nigeria†	77
Togo	62

The West African countries surveyed included the above highlighted 8 countries and 6 additional countries: Benin, Gambia, Ghana, Liberia, Senegal, and Sierra Leone. Note: Countries are arranged in alphabetical order. Bold figure – indicates the lowest capacity. † – mpox-affected countries.

Interpretation – Of the three mpox-affected West African countries, this suggests poor logistics (vaccine access) readiness capacity in two (66.7%) countries, Côte d'Ivoire and Nigeria, with both reporting a level of 77% each, and a weak diagnostics capacity in one (33.3%) country, Côte d'Ivoire. Additionally, some of the specific problems highlighted above have been reported among the West African countries, in which a majority, including Cote d'Ivoire, Guinea-Bissau, and Cabo Verde were found to lack functional cold chains, as well as the necessary supply chain and logistics mechanisms for ensuring uninterrupted access to mpox vaccines, as they become available.

Role of the West African Health Organization (WAHO) and Other Actors

As a strategic response to some of these issues, WAHO is putting efforts in place to hold the Conference of the Heads of States of Economic Community of West African States (ECOWAS) on mpox to identify and harmonize medical countermeasures (MCM), workforce training, and research needs for controlling mpox in the region.

By collaborating with the Africa Centres for Disease Control and Prevention (Africa CDC), World Health Organization African Region (WHO AFRO) and the private sector, WAHO seeks to work with individual countries to come up with an expression of the total needs for mpox response.

This will include making a pull procurement for vaccines, therapeutics, and diagnostics amongst other essential commodities, which could result in having timely and cheaper health emergency commodities.

To date, ongoing response interventions provided by WAHO to the ECOWAS Member States include (1) regional tabletop simulation exercise to strengthen preparedness and response to mpox, (2) online coordination meeting with heads of National Public Health Institutes (NPHIs) on mpox, (3) regional training workshop for laboratory specialists to strengthen detection and genomic sequencing of mpox in West Africa with Pasteur Institute collaboration, and (4) supply of laboratory reagents to all national reference laboratories of 15 ECOWAS Member States in collaboration with Africa CDC, WHO AFRO and the private sector.

Importantly, drawing on our collective experience from responses to previous public health emergencies, including but not limited to Ebola, mpox, and the COVID-19 pandemic, WAHO recognizes the need for a system-wide comprehensive, concerted, and coordinated response to mpox that is centred on pragmatism and equity principles. This is necessary because it is crucial to balance "what is known" and "what is available" with "what is feasible" to achieve "what is desired" without widening existing health inequities or creating new ones.

Box S1. Short-term pragmatism and equity considerations for mpox at the national, regional, or continental level [16-20].

a. Pragmatism

Epistemological paradigm

Assess and monitor the:

- mpox epidemiological and virological profile leveraging existing surveillance systems
- vulnerability and capacities for response to the disease through documents review, peer discussions, and literature search
- risk of the disease transmission based on its likelihood and impact using standardized methodology such as the World Health Organization (WHO) Strategic Toolkit for Assessing Risks (STAR), or as provided in the global strategic and technical guidance for the disease
- response strategies and actions for the disease, in line with regional, continental, and global guidelines and prioritize them based on their usability

Approach to change

Identify and implement all:

- locally relevant mpox response strategies and actions based on their feasibility

b. Equity

Process

Identify and classify the:

- geographical areas based on the mpox risk level for tailored response actions
- populations based on the disease risk level for tailored response actions
- geographical areas affected by disease outbreaks other than the current disease for tailored response actions
- populations concurrently affected by disease outbreaks other than mpox for tailored response actions

Outcome

Evaluate and address disparities in:

- access to medical countermeasures for mpox among high-risk geographical areas (based on the disease endemicity), vulnerable geographical areas (based on multi-disease outbreak burden and socioeconomic level), and underserved communities (based on disparities in social determinants)
- access to medical countermeasures among at-risk and vulnerable populations
- clinical outcomes of the disease (e.g., hospitalization, admission to intensive care unit, death) among at-risk populations, vulnerable populations, and underserved communities

Box S2. The West African Health Organization (WAHO) key strategic response plan and recommendations for mpox in the Economic Community of West African States (ECOWAS) region. This was based on the critical strategies and actions determined following a holistic rapid situational assessment of the regional disease risk, health systems vulnerability, and available capacity through review of government or institutional reports (surveillance data), literature search, and discussions among the region's Health Ministers between August and September 2024 [16,21-22].

a. Key strategic response plan of WAHO for mpox

1. Strengthen regional coordination.
2. Improve epidemiological surveillance and research.
3. Strengthen diagnostic and genomic sequencing capacities at the laboratory level.
4. Increase risk communication and community involvement.
5. Reinforce cross-border surveillance.
6. Expand access to vaccines and products for management.
7. Mobilize related resources to implement interventions.

b. Key recommendations of WAHO to ECOWAS Health Ministers for mpox

1. *Enhancing public awareness*: we recommend launching public awareness campaigns focused on educating communities about mpox. Clear and accessible messaging on transmission prevention, symptoms, and when to seek medical help is essential. Empowering populations with knowledge is a key step in preventing the further spread of mpox.
2. *Strengthening surveillance and response*: we recommend strengthened surveillance systems to detect and respond to mpox cases promptly.
3. *Capacity building in laboratory testing*: we emphasize that the capacity of the national and regional laboratories in management and testing of mpox samples be determined and support offered where gaps are identified.
4. *Strengthening cross-border collaboration*: given the cross-border nature of disease transmission, we strongly encourage enhanced cooperation among neighbouring countries. Coordinated efforts in surveillance, data sharing, and joint outbreak response will be instrumental in containing the spread of mpox across borders.
5. *Training healthcare workers*: we emphasize the importance of continuous capacity building for healthcare workers. Training on case detection, case management and isolation, and general infection control will equip healthcare systems to manage cases effectively and prevent hospital-acquired infections.

Global health architecture reform

The repetitive pattern of "promising and failing" for global health goals such as the 1978 Health for All by 2000 Alma Ata Declaration, the 2000 Millenium Development Goals (MDGs) by 2015, and the slow and uneven progress of the 2015 Sustainable Development Goals (SDGs) by 2030 [23-25], suggests that the global health architecture needs to be carefully examined and systematically reformed.

The increasing negative consequences of these failures, particularly in low- and middle-income countries (LMICs) are not only worsening outcomes at the health level (e.g., morbidity and mortality), social level (multidimensional poverty), economic level (health costs), and technical level (health systems fragmentation), but they are also causing far significant impact at the personal level, given the human component of the architecture. *By overpromising and underdelivering, trusts are eroded, collaborations are questioned, and commitments are weakened*. The neglect of mpox, increased fragmentation of national and regional health systems from donor-dependent vertical global health programmes, and failure of the COVID-19 Vaccines Global Access (COVAX) to deliver on its promises [26-28], despite countries' agreements to uphold the principle of equity and moral rule of engagement, further paint a clearer picture of why this reform need to begin now and with bold actions.

A good example of this unfairness can be seen is through the Pushkaran and colleagues' critical analysis of COVAX–a multistakeholder initiative launched in April 2020, and led by the Vaccine Alliance (Gavi), Coalition for Epidemic Preparedness Innovations (CEPI), WHO, and United Nations Children's Fund (UNICEF). Although this was considered an exemplary global health model, it fell short of its good intentions and main goal to facilitate equitable and timely access to vaccines during the COVID-19 pandemic [28]. Shockingly, in January 2021, this model resulted in 60% of the total vaccine pool being accessed in high-income countries (HICs)–where only 16% of the world population reside–, meaning that LMICs were only left with the remaining 30%. This act of vaccine nationalism made COVAX deliver below its target by 32% (of 1.4 billion vaccine doses pledged) as of December 2021, leaving populations in LMICs vulnerable to the pandemic. By design, this

model was reported to have violated its equity principle through its proportional allocation mechanism of vaccine distribution instead of a need-based mechanism and disproportionate vaccination target (20% for LMICs, and 50% for HICs) [28].

Specifically, in terms of knowledge generation practices, the same study and another study by Usher and colleagues showed that this was heavily in favour of HICs, such as the UK influence in the proposition of the "Optional Purchase Agreement" that allowed only HICs to have a flexible choice for vaccines, though at the expense of higher cost [28-29]. Also, concerning its leadership structure, it was reported to lack an inclusive global governance mechanism and transparent decision-making [28-29]. The same weaknesses have also been reported in a similar global health model, the Pandemic Fund, launched in 2022 [30-31]. This has also been criticized for similar leadership issues and suboptimal support for emergency response, a capacity that is significantly underdeveloped and most needed in LMICs, where health systems are increasingly strained due to the disproportionate burden of epidemic- and pandemic-prone diseases like mpox [30-32]. Perhaps this is the time for the global health community to consider approaching this reform through a Learning Health System and Implementation Science lens to make the architecture more fit-for-purpose for all.

With long-term considerations of both equity as a process and as an outcome placed at the forefront of this reform, efforts must be those that seek to spur a paradigm shift in the systems and processes of knowledge generation, learning, leadership, workflow, and funding within the global health architecture, in which infectious disease emergency response is embedded, from its current state to an equitable and sustainable one (**Box S3**).

In the spirit of self-determination, it is expected that with this this emerging shift, longstanding public health problems in Africa, such as mpox and Lassa would be actively championed, domestically funded, and internationally supported, as a regional-led agenda, at the level of global health actors like WHO AFRO, with significant collaborations and inputs from other health and non-health actors, including Africa CDC, WAHO, and all the Member States.

Regional and national health systems strengthening

Steering this renewed vision also makes it very important for lead health institutions in the African region to question their "positions" and "contributions" in the global health architecture. This self-reflection is rooted in the popular maxim of equity that "*he who seeks equity must do equity*" [33]. These institutions must critique the weaknesses in their health systems and processes that reinforce the systemic barriers in the global health architecture. Raising important equity-focused questions first would set the stage for more thought-provoking questions. This should begin by asking how the external funding dependency of African health institutions has affected their decision-making power in global health agenda setting. And what have been the contributions of these institutions to global public goods for health?

Nevertheless, it has become increasingly evident that the region must find creative ways to become financially self-reliant in the event of any major infectious disease emergency. For example, Nonvignon and colleagues showed that external funding accounted for 10% of total health spending in 2020, with the highest rate in Eastern Africa (27%), followed by Central Africa (22%) and West Africa (12%) [34]. But this funding has remained stagnant and even declined, in some instances, since 2020 due to economic contractions occasioned by the COVID-19 pandemic [34]. Similarly, a WHO report found that half of the African countries had 20% of their current health expenditures funded through external funding between 2012 and 2020, suggesting a chronic state of donor dependency [35].

This current financial landscape, which is similar to the substantial reliance of key lead health institutions, including some NPHIs, WAHO, and the Africa CDC and its parent body (the African Union), on external resources for public health emergency responses and regional integration [8,26-37], is unsustainable. Fair taxation, strategic public-private partnerships, and domestic fundraising campaigns are all high-yield, proven approaches that should be sustainably implemented at scale [38-40]. These are crucial for financial autonomy, without which there can be no health systems strengthening.

More so, evidence that 87% of the voluntary contributions to WHO, which accounts for 80% of its total budget that are strictly tied to specific global health programs for a particular timeframe [41]. With the current weak state of most African health systems despite decades-long donor dependency, and the rapidly evolving digital landscape, strengthening regional and national health systems is inevitable to improve health security and human development. Learning from past health systems strengthening challenges, this effort must start from down to the top – local to national at the government level, community to health facility at the infrastructure level, rural to urban at the community level, and primary to tertiary at the health facility level.

More so, achieving this would require: 1) mandating a comprehensive revision of critical health policies, including training curriculums and employment to improve and standardize training, practice, and renumeration for high health workforce retention, and 2) a once-and-for-all commitment to the Abuja Declaration on national health expenditure target (15% of gross domestic product) for improved universal health coverage and public health emergency response outcomes [40,42]. This is feasible and has the potential to support local priorities, assure national sovereignty, and accelerate national development as the key pathways to sustainable development, as demonstrated with a regional vaccine access model, the Africa Vaccine Acquisition Trust (AVAT), which was reported to have performed better in facilitating timely and equitable vaccine access in the region compared to COVAX [43-44].

Undoubtedly, by ensuring the financial autonomy of these lead health institutions that is grounded in transparent and accountable domestic resource mobilization, improved auditing, and dedicated funding mechanism (e.g., infectious disease emergency fund), coupled with proactive and resilient health systems, the neglect of diseases of public health importance like mpox could be prevented and their associated morbidity and mortality reduced substantially. In the longer term, all things being equal, these changes should translate into equitable decision-making for agenda setting and contributions to global public goods for health within the global health architecture, thereby providing a strong momentum for its effective and timely reform.

Similar to outbreak and epidemic response (secondary pandemic prevention), increased commitments and investments must also be directed towards building capacities for pandemic prevention, focusing on risk at the source (i.e., primary pandemic prevention) to minimize human exposures to infectious zoonotic diseases. To reduce this risk, ecological countermeasures to prevent pathogen spillover from animals to humans in wildlife natural forest habitats and rural areas, sustained spread among human populations in rural and suburban/urban areas during outbreaks or epidemics, and their possible propagation into pandemics through international spread in multiple countries, must be developed, implemented, and scaled up. This understanding has now culminated in what has been described as "ecological surveillance", which emphasizes land use and sociobehavioural interventions that protect or restore wildlife habitat and mitigate wildlife-human interactions to reduce the risk of pathogen spillover, focusing on its upstream factors [45].

In the context of mpox, this must quickly be advanced into One Health ecological surveillance for practical high-yield overarching strategies and actions. Through this approach, these interventions must be aimed to manage the: 1) natural habitat of potential mpox reservoir hosts (e.g., squirrels, rats) and intermediate hosts (apes, monkeys) by protecting their forages (i.e., where they eat) and roosts (where they sleep), 2) natural habitat-rural areas interface to prevent or minimize reservoir host-livestock/pet animal and human interactions by replanting trees and vegetations and restoring water resources around forages and roosts, and 3) suburban/urban environments to reduce the long-term risk of livestock and human exposures to the disease by increasing risk communication and community engagement on its reservoir hosts conservation and risk mitigation among hunters, farmers, urban planners, house builders, community leaders, and home owners [45-46].

Lastly, these primary pandemic interventions should be complemented with pandemic preparedness or secondary pandemic prevention interventions, including ongoing collaborative surveillance that leverages genomic sequencing, serological testing, and environmental sampling to advance understanding on MPXV transmissions in natural habitats, reservoir hosts, intermediate hosts, livestock/pets, and human populations,

identifying its geographical hotspots for potential outbreaks. This should also involve the translation of surveillance evidence and insights into policies on hunting, farming, urban planning, home building, community, home protection, and mpox preparedness resource allocation. Research and development for novel diagnostics, vaccines, and therapeutics for enhanced community protection during outbreaks, epidemics or pandemics, is also imperative to achieve and sustain progress. This integrated response approach is critical to help reduce the burden of zoonotic and human-human transmissions that go undetected, allowing reliable infectious disease emergency early warning, situational awareness, risk prediction capabilities for improved preparedness and response.

Box S3. Long-term pragmatism and equity considerations for mpox and other infectious diseases at the global health architecture level.

a. Equity

Process

Identify and shift the:

- knowledge systems for global evidence generation from pro Global North to pro Global North and Global South
- learning systems for global evidence improvement from top-down (globalized) to bottom-top (glocalized) approach
- leadership systems for governance from one that is disproportionately represented by the Global North to a fairly representative one with the inclusion of the Global South
- workflow processes for political and technical activities from colonial and patriarchal-based worldviews to those decolonial and egalitarian in nature
- funding systems with assessed contributions from unpredictable to predictable and voluntary contributions from earmarked to flexible

Outcome

Evaluate and address disparities in:

- generation of global evidence by stakeholder representativeness (based on Global North and Global South diversity, inclusion, and equity) and language variability (based on the United Nations official languages: Arabic, Chinese, English, French, Russian, and Spanish) among global health institutions
- improvement of global evidence by bottom-top approach (based on how contextualized knowledge on evidence implementation from local level is fed back into the global evidence base) among global health institutions
- governance by leadership representativeness (based on Global North and Global South diversity, inclusion, and equity) among global health institutions
- political and technical activities by systems of oppression reduction and power and privilege balance (based on decolonial and egalitarian worldviews) among global health institutions
- assessed contributions (based on predictability) among national governments and voluntary contributions (based on flexibility) among global health institutions

b. Pragmatism

Epistemological paradigm

Assess and monitor the:

- evidence of WHAT global health architecture systems and processes work (based on an equitable global health architecture components) for infectious disease emergency response, HOW it worked (based on implementation strategies and mechanisms), and WHY it worked (based on implementation determinants and contextual factors)

Approach to change

Identify and implement all:

- feasible global health architecture systems and processes for mpox response based on their demonstrated added value in terms of access to medical countermeasures and clinical outcomes

Box S4. Implementation priorities for mpox.

1. *Clear coordination mechanism*: coordination mechanisms for all actors providing strategic and technical support should be integrated vertically (i.e., among subnational, national, regional, continental, and global health actors with identification of the lead institution) and horizontally (i.e., among other sectors' actors with identification of the facilitating institution) with clear roles and responsibilities to ensure efficient, transparent, and accountable mpox response. In the longer term, this will help minimize duplication of efforts and optimize resource mobilization and allocation. To this end, the West African Health Organization (WAHO) has organized the first emergency meeting for Health Ministers in West Africa on Aug 22, 2024, to share information on the epidemiological situation and discuss immediate public health measures to be taken both at the regional and national levels [22]. The Economic Community of West African States (ECOWAS) Ministers of Health have ended their meeting with several recommendations to rapidly control the mpox outbreak. Moreover, WAHO has established a regular meeting of national public health institutes (NPHIs) to enhance information sharing, learning, and dissemination of best practices.
2. *Cohesive capacity building*: capacity building should be provided for health workers at the subnational, national, regional, and continental levels through pre-identified facilitating platforms to ensure trainings on mpox are prioritized and jointly delivered, where possible in line with stipulated standards to promote consistency of practice and access to robust health information. In the longer term, this will help contribute to duplicated efforts reduction and resource mobilization and allocation optimization [18]. To this end, WAHO has supported the training of national One Health coordination mechanisms on mpox and the training of laboratory specialists for mpox diagnostics and genomic sequencing. In addition, capacity building efforts have included training of points of entry (POE), infection prevention and control (IPC), and case management experts.
3. *Consistent risk assessment*: risk assessment for the disease should be conducted using the same standardized methodology at the subnational, national, regional, and continental levels based on global best practices such as the World Health Organization (WHO) Strategic Toolkit for Assessing Risks (STAR) [16], to ensure adequate priority setting, resource mobilization, and response. In the longer term, this will help accelerate progress toward its control. To this end, WAHO continues to assess mpox risk consistent with the WHO guideline based on changing contexts for enhanced situational awareness of the outbreak and response interventions.
4. *Unified case reporting*: complete data on cases should be reported by countries at the same timeline to the regional, continental, and global surveillance databases using a common case definition (e.g., the WHO case definition) to provide reliable evidence to better estimate the true burden of the disease for adequate response and real-world health impact. In the longer term, this will help minimize duplication of efforts and accelerate progress toward its control. To this end, WAHO continues to engage with its Member States and advocate for the use of the WHO's mpox case definition and plans to support the printing and dissemination of case definition handouts to ensure regional representativeness of epidemiological data.
5. *Locally led and internationally supported research*: research should align with countries' contexts and priorities, promote the bidirectional transfer of knowledge, and be conducted through a multidisciplinary lens (e.g., One Health) to better characterize the epidemiological, virological, and clinical patterns of the disease, the effectiveness of its response strategies and actions, and promote capacity building. For example, the recent establishment of the Mpox Research Consortium (MpoxReC) is a bold step towards this vision [47]. Nonetheless, a strong accountability mechanism should be created to ensure that the principles of equitable global health partnerships are upheld. In the longer term, this will help in its effective and timely control. To this end, WAHO is committed to supporting its Member States in providing strong political commitments and investments towards the MpoxReC's research agenda in alignment with national priorities.
6. *Commodity and vaccine security:* medical countermeasures should be procured through a pooled and pull mechanism to ensure the continued availability of clinical and laboratory commodities and vaccines needed to maintain the ability of countries to diagnose cases of mpox and prevent avoidable morbidity and mortality from the disease, particularly among the at-risk and vulnerable populations. In the longer term, this will help contribute to effective and timely control of the mpox outbreak. To this end, WAHO is committed to working collaboratively with Africa Centres for Disease Control and Prevention (CDC) and WHO Regional Office for Africa (AFRO) to identify and define its role and other regional institutions in supporting the commodity and vaccine procurement and distribution strategy of the Mpox Continental Preparedness and Response Plan 2024-2025 [48].
7. *Integrated monitoring and evaluation*: monitoring and evaluation for the disease response strategy should be integrated into its control and elimination framework to ensure that response actions are comprehensive, timely, and adaptable based on changing contexts. In the longer term, this will help promote accountability and accelerate progress toward its control. To this end, WAHO will be contributing to the monitoring and evaluation framework of the Mpox Continental Preparedness and Response Plan 2024-2025 [48].

Monitoring, Evaluation, and Learning

Both recommendations and implementation priorities as highlighted in the main text must be underpinned by a monitoring, evaluation, and learning (MEL) system for tracking the progress and assessing the impact of the response strategy in real-time for data-driven adjustments based on changing circumstances, while learning from the processes and results to improve future outbreak responses.

This system must comprise of these requirements based on best practices: 1) plan and standard operating procedure, 2) dedicated fund, 3) team, 4) focal person, 5) logic model, 6) logframe, 7) data dictionary, 8) database, 9) age/gender/location disaggregated data, and 10) dissemination plan.

Although this is not an exhaustive list of evidence-based strategies and metrics, adopting or adapting them with fidelity could help in mounting a comprehensive, concerted, and coordinated mpox response towards breaking silos, standardizing strategy and action plans, optimizing scarce resources, reducing health disparities, and improving transparency and accountability for high impact.

Finally, we recognize that this process is more complex than its simple presentation in this paper. Therefore, our recommendations and implementation priorities for mpox response should be interpreted and considered within its limited context due to the rapid nature of this analysis, the evolving nature of the outbreak in the region, and the potential for information bias.

Table S3. Monitoring, evaluation, and learning (MEL) system for mpox response implementation per WHO guidance and Implementation Science [49-56].

<table>
<tr><th>WHO Strategy and Action</th><th>Monitoring</th><th>Evaluation</th><th>Learning</th></tr>
<tr>
<td>Mpox
Monitoring goal
1. Enhance response effectiveness
2. Enhance response efficiency
3. Enhance response equity
Evaluation goal
4. Improve response outcomes
5. Improve response impact
6. Improve response implementation
Learning goal
7. Increase response adaptation
8. Increase response evidence base
9. Increase knowledge translation</td>
<td>Aim
To track the progress of response activities in real-time for continuous feedback to inform decision-making
Key element
▪ Key performance indicators (KPI) rates: surveillance coverage, case detection, vaccination coverage, public health and social measures uptake, treatment outcomes, and community engagement + KPI for scale up: credibility, observability, relevance, relative advantage, easy to install and understand, compatibility, and testability of strategy
▪ Data management processes: weekly-monthly integration and reporting of data from health facilities, laboratories, community surveys, and digital health platforms
▪ Regular monitoring reviews: biweekly-monthly data analysis and actionable insights generation on KPI rates</td>
<td>Aim
To assess the impact of response strategy, successes, challenges, and lessons learned for improvement
Key element
▪ Outcome and impact assessments rates: incidence, outbreak expansion, morbidity and mortality, and healthcare access
▪ Mid-term and final evaluations: analysis of MEL goals for course correction and success tracking
▪ Intra-action and after-action reviews: critiquing of response during and after emergency to synthesize lessons and best practices
▪ Community feedback mechanisms: mixed-methods assessment of perception on public messaging, services access, and satisfaction</td>
<td>Aim
To foster transparency, local adaptation, and trust for high impact
Key element
▪ Real-time dashboards: multi-stakeholder visualization of MEL results and actionable insights
▪ Regular stakeholder reporting: KPI /outcomes/impact, lessons, and adjustments reporting
▪ Public accountability: results sharing via publication, press briefing, and other media
▪ Adaptive management: action and resource allocation adjustment</td>
</tr>
<tr><td colspan="4"></td></tr>
<tr>
<td>Strengthened collaborative surveillance and detection</td>
<td>Percentage of: 1) states with ongoing mpox surveillance systems, 2) suspected mpox cases and events investigated within 24 hours of reporting, 3) contacts of mpox cases traced and monitored, 4) suspected mpox cases reported from local to national within 24 hours, 5) suspected mpox cases tested, 6) confirmed mpox cases sequenced</td>
<td rowspan="5">Outcomes
Transmission rate: percentage of population at risk with suspected or confirmed mpox
Severity rate: percentage of confirmed mpox cases among population at risk hospitalized
Expansion rate: percentage of previously unaffected states reporting new mpox cases
Impact
Morbidity rate: percentage of total population with mpox
Mortality rate: percentage of total population with mpox that died
Implementation
Reach: percentage of communities at risk engaged and supported on mpox response
Fidelity: percentage of states implementing core mpox interventions
Maintenance: percentage of states implementing core mpox interventions throughout the outbreak period</td>
<td rowspan="5">Contextualization
Contextual fit: percentage of states with optimal acceptability and preference for mpox strategy
Feasibility: percentage of states with optimal adoption and resource availability for mpox strategy
Barriers or facilitators: percentage of states reporting barriers or facilitators to core mpox interventions
Evidence base
Repository: percentage of states with an open repository on mpox response
Publication: percentage of states with one publication on mpox response
Knowledge translation
Translation: percentage of states integrating adaptations into policy and lessons into SOPs and workflows</td>
</tr>
<tr>
<td>Enhanced community protection</td>
<td>Percentage of: 1) individuals in high-risk areas with mpox interventions uptake, 2) states implementing mpox infection prevention and control (IPC) and water, sanitation and hygiene</td>
</tr>
<tr>
<td>Safe and scalable care</td>
<td>Percentage of: 1) health facilities with trained staff on mpox case management, 2) health facilities with care plan, standard operating procedures (SOPs), guidelines, and clinical care pathways, and IPC for mpox</td>
</tr>
<tr>
<td>Equitable access to medical countermeasures</td>
<td>Percentage of: 1) states with active mpox outbreaks with access to vaccines and therapeutics, 2) states with mpox outbreaks where communities accessed training, finance, and supplies for outreach and engagement, 3) percentage of needed vaccine doses distributed, 4) target population vaccinated against mpox</td>
</tr>
<tr>
<td>Emergency coordination</td>
<td>Percentage of: 1) functional mpox coordination structures established in states</td>
</tr>
</table>

References

1. Sheikh K, Peters D, Agyepong IA, Abimbola S, Ghaffar A, Swaminathan S. Learning is a means to progress and empowerment for health systems. BMJ Glob Health. 2022;7(Suppl 7):e010572.

2. Rutter H, Savona N, Glonti K et al. The need for a complex systems model of evidence for public health. Lancet. 2017;390(10112):2602-2604.

3. Mitjà O, Ogoina D, Titanji BK et al. Monkeypox. Lancet. 2023;401(10370):60–74.

4. Laurenson-Schafer H, Sklenovská N, Hoxha A et al. Description of the first global outbreak of mpox: an analysis of global surveillance data. Lancet Glob Health. 2023;11(7):e1012–e1023.

5. World Health Organization (WHO). Mpox: key facts. WHO; 2024. Available: https://www.who.int/news-room/fact-sheets/detail/mpox. Accessed: 27 Aug 2024.

6. Gao S, Zeng Z, Zhai Y et al. Driving effect of multiplex factors on Mpox in global high-risk region, implication for Mpox based on one health concept. One Health. 2023;17:100597.

7. Rivers C, Watson C, Phelan AL. The resurgence of mpox in Africa. *JAMA*. 2024;332(13):1045–1046.

8. Adetifa I, Muyembe JJ, Bausch DG, Heymann DL. Mpox neglect and the smallpox niche: a problem for Africa, a problem for the world. Lancet. 2023;401(10390):1822–1824.

9. Moyo E, Musuka G, Murewanhema G, Moyo P, Dzinamarira T. Monkeypox outbreak: a perspective on Africa's diagnostic and containment capacity. Int J Infect Dis. 2022;123:127–130.

10. Reynolds MG, Doty JB, McCollum AM, Olson VA, Nakazawa Y. Monkeypox re-emergence in Africa: a call to expand the concept and practice of One Health. Expert Rev Anti Infect Ther. 2019;17(2):129–139.

11. Africa Centres for Disease Control and Prevention (Africa CDC). Africa CDC and Bavarian Nordic partner to boost mpox vaccine production in Africa. Africa CDC; 2024. Available: https://africacdc.org/news-item/africa-cdc-and-bavarian-nordic-partner-to-boost-mpox-vaccine-production-in-africa/. Accessed: 26 Aug 2024.

12. Adepoju P. Mpox declared a public health emergency. Lancet. 2024;404(10454):e1-e2.

13. Taylor L. First mpox vaccines arrive in Africa as officials work "blindly" to contain outbreaks. BMJ. 2024;386:q1897.

14. Chikezie NC, Shomuyiwa DO, Okoli EA et al. Addressing the issue of a depleting health workforce in sub-Saharan Africa. Lancet. 2023;401(10389):1649–1650.

15. United Nations Children's Fund (UNICEF). Multiple and simultaneous epidemics on the rise in West and Central Africa. UNICEF; 2022. Available: https://www.unicef.org/wca/press-releases/multiple-and-simultaneous-epidemics-rise-west-and-central-africa. Accessed: 26 Aug 2024.

16. World Health Organization (WHO). Strategic toolkit for assessing risk: a comprehensive toolkit for all-hazards health emergency risk assessment. WHO; 2021. Available: https://iris.who.int/bitstream/handle/10665/348763/9789240036086-eng.pdf?sequence=1. Accessed: 26 Aug 2024.

17. Lokossou VK, Awori AS, Fatimehin V et al. Assessing mpox epidemic readiness status in ECOWAS region: strengths, gaps, and recommendations for an improved response. Pan Afr Med J. 2025;50(1):2.

18. Smith J, Boro E, Kwong EJL, Schmidt-Sane M. Resisting unchecked pragmatism in global health. Lancet Glob Health. 2023;11(8):e1176–e1177.

19. Network of Schools of Public Policy, Affairs, and Administration (NASPAA). Taskforce on diversity, inclusion, and equity: toward a comprehensive framework and action plan September 2020. NASPAA; 2020. Available: https://www.naspaa.org/sites/default/files/docs/2020-12/21%20DEI%20Task%20Force%20Final%20Report%2010-5-20.pdf. Accessed: 26 Aug 2024.

20. Braveman P, Gruskin S. Defining equity in health. J Epidemiol Community Health 2003;57(4):254–8.

21. West African Health Organization (WAHO). Emergency bulletin of mpox in West Africa: mpox situation report N3 03 Sep 2024. WAHO; 2024. Available: https://www.wahooas.org/web-ooas/en/publications-et-recherches/bulletins-epidemiologiques. Accessed: 07 Sep 2024.

22. West African Health Organization (WAHO). Emergency meeting on mpox: press release. WAHO; 2024. Available: https://www.wahooas.org/web-ooas/en/actualites/emergency-meeting-mpox-press-release. Accessed: 26 Aug 2024.

23. Rifkin SB. Alma Ata after 40 years: Primary Health Care and Health for All-from consensus to complexity. BMJ Glob Health. 2018;3(Suppl 3):e001188.

24. Lomazzi M, Borisch B, Laaser U. The Millennium Development Goals: experiences, achievements and what's next. Glob Health Action. 2014;7:23695.

25. Liu Y, Du J, Wang Y, Cui X et al. Overlooked uneven progress across sustainable development goals at the global scale: Challenges and opportunities. Innovation (Camb). 2024;5(2):100573.

26. Spicer N, Agyepong I, Ottersen T, Jahn A, Ooms G. 'It's far too complicated': why fragmentation persists in global health. Global Health. 2020;16(1):60.

27. Pushkaran A, Chattu VK, Narayanan P. A critical analysis of COVAX alliance and corresponding global health governance and policy issues: a scoping review. BMJ Glob Health. 2023;8(10):e012168.

28. Usher AD. A beautiful idea: how COVAX has fallen short. Lancet. 2021;397(10292):2322-2325.

29. Boyce MR, Sorrell EM, Standley CJ. An early analysis of the World Bank's Pandemic Fund: a new fund for pandemic prevention, preparedness and response. BMJ Glob Health. 2023;8(1):e011172.

30. Ndembi N, Dereje N, Nonvignon J et al. Financing pandemic prevention, preparedness and response: lessons learned and perspectives for future. Global Health. 2024;20(1):65.

31. Torres Munguia JA, Badarau FC, Diaz Pavez LR, Martinez-Zarzoso I, Wacker KM. A global dataset of pandemic- and epidemic-prone disease outbreaks. Sci Data. 2022;9(1):683.

32. Chafee Z. Coming into Equity with Clean Hands. II. Michigan Law Review. 1949;47(8):1065-96.

33. Nonvignon J, Soucat A, Ofori-Adu P, Adeyi O. Making development assistance work for Africa: from aid-dependent disease control to the new public health order. Health Policy Plan. 2024 Jan 23;39(Supplement_1):i79-i92.

34. World Health Organization (WHO). WHO African region health expenditure atlas 2023. WHO;2023. Available: https://www.afro.who.int/publications/who-african-region-health-expenditure-atlas-2023-0. Accessed: 22 Oct 2024.

35. Apeagyei AE, Lidral-Porter B, Patel N et al. Financing health in sub-Saharan Africa 1990-2050: Donor dependence and expected domestic health spending. PLOS Glob Public Health. 2024;4(8):e0003433.

36. Institute for Security Studies (ISS). Financial independence is key to stronger AU partnerships. ISS;2023. Available: https://issafrica.org/iss-today/financial-independence-is-key-to-stronger-au-partnerships. Accessed: 22 Oct 2024.

37. Stapel S, Söderbaum F. European foreign aid to regional organisations in Africa: bullies, overseers, micromanagers and samaritans. Third World Q. 2023;44(8):1699-1717.

38. Reeves A, Gourtsoyannis Y, Basu S, McCoy D, McKee M, Stuckler D. Financing universal health coverage--effects of alternative tax structures on public health systems: cross-national modelling in 89 low-income and middle-income countries. Lancet. 2015;386(9990):274-80.

39. Fanelli S, Salvatore FP, De Pascale G, Faccilongo N. Insights for the future of health system partnerships in low- and middle-income countries: a systematic literature review. BMC Health Serv Res. 2020;20(1):571.

40. Alilio M, Hariharan N, Lugten E et al. Strategies to promote health system strengthening and global health security at the subnational level in a world changed by COVID-19. Glob Health Sci Pract. 2022;10(2):e2100478.

41. World Health Organization (WHO). How WHO is funded. WHO; 2025. Available: https://www.who.int/about/funding. Accessed: 03 Jan 2025.

42. World Health Organization (WHO). WHO African region health expenditure atlas 2023. WHO;2023. Available: https://www.afro.who.int/publications/who-african-region-health-expenditure-atlas-2023-0. Accessed: 22 Oct 2024.

43. Ndembi N, Dereje N, Nonvignon J et al. Financing pandemic prevention, preparedness and response: lessons learned and perspectives for future. Global Health. 2024;20(1):65.

44. World Health Organization (WHO). Supply bottleneck, financial challenges fuel delays in Africa's COVID-19 vaccine rollout. WHO; 2021. Available: https://www.afro.who.int/news/supply-bottleneck-financial-challenges-fuel-delays-africas-covid-19-vaccine-rollout#:~:text=19%20vaccine%20rollout-,Supply%20bottleneck%2C%20financial%20challenges%20fuel%20delays,Africa's%20COVID%2D19%20vaccine%20rollout&text=Brazzaville%20%E2%80%93%20A%20blockage%20on%20supplies,continent's%20rollout%20later%20this%20year. Accessed: 22 Oct 2024.

45. Plowright RK, Ahmed AN, Coulson T et al. Ecological countermeasures to prevent pathogen spillover and subsequent pandemics. Nat Commun. 2024;15(1):2577.

46. Gessain A, Nakoune E, Yazdanpanah Y. Monkeypox. N Engl J Med. 2022;387(19):1783-1793.

47. Nachega JB, Sam-Agudu NA, Ogoina D et al. The surge of mpox in Africa: a call for action. Lancet Glob Health. 2024;12(7):e1086–e1088.

48. Africa Centres for Disease Control and Prevention (Africa CDC). Mpox continental preparedness and response plan for Africa. Africa CDC; 2024. Available: https://africacdc.org/download/mpox-continental-preparedness-and-response-plan-for-africa/. Accessed 06 Sep 2024.

49. Damschroder LJ, Reardon CM, Widerquist MAO, Lowery J. The updated Consolidated Framework for Implementation Research based on user feedback. Implement Sci. 2022;17(1):75.

50. World Health Organization (WHO). Nine strategies for developing a scaling-up strategy. WHO;2010. Available: https://iris.who.int/bitstream/handle/10665/44432/9789241500319_eng.pdf?sequence=1. Accessed: 06 Sep 2024.

51. Ogunyemi KO, McNabb S, Lokossou V, Sogbossi LS, Nyenswah T, Ohuabunwo C. Developing a new pragmatic tool for assessing contextual fit and feasibility of evidence-based interventions towards effective implementation in global health. BMJ Glob Health. 2025;10(4):e015931.

52. Proctor E, Silmere H, Raghavan R et al. Outcomes for implementation research: conceptual distinctions, measurement challenges, and research agenda. Adm Policy Ment Health 2011; 38:65-76.

53. Glasgow RE, Harden SM, Gaglio B et al. RE-AIM planning and evaluation framework: adapting to New Science and Practice With a 20-Year Review. Front Public Health. 2019;7:64.

54. Chambers DA, Norton WE. The adaptome: advancing the science of intervention adaptation. Am J Prev Med. 2016;51(4 Suppl 2):S124-S131.

55. Bochner AF, Makumbi I, Aderinola O et al. Implementation of the 7-1-7 target for detection, notification, and response to public health threats in five countries: a retrospective, observational study. Lancet Glob Health. 2023;11(6):e871-e87.

56. World Health Organization (WHO). Mpox global strategic preparedness and response plan: draft. WHO; 2024. Available: https://www.who.int/publications/m/item/mpox-global-strategic-preparedness-and-response-plan. Accessed: 26 Aug 2024.
